# The ‘Threat of Scream’ paradigm: a tool for studying sustained physiological and subjective anxiety

**DOI:** 10.1038/s41598-020-68889-0

**Published:** 2020-07-27

**Authors:** Morgan Beaurenaut, Elliot Tokarski, Guillaume Dezecache, Julie Grèzes

**Affiliations:** 1 Laboratoire de Neurosciences Cognitives et Computationnelles, ENS, PSL Research University, INSERM, Département d’études Cognitives, Paris, France; 2 Department of Experimental Psychology, Division of Psychology and Language Sciences, University College London, London, UK; 3 Université Clermont Auvergne, CNRS, LAPSCO, Clermont-Ferrand, France

**Keywords:** Human behaviour, Cognitive neuroscience, Emotion, Stress and resilience, Neuroscience, Psychology, Anxiety

## Abstract

Progress in understanding the emergence of pathological anxiety depends on the availability of paradigms effective in inducing anxiety in a simple, consistent and sustained manner. The Threat-of-Shock paradigm has typically been used to elicit anxiety, but poses ethical issues when testing vulnerable populations. Moreover, it is not clear from past studies whether anxiety can be sustained in experiments of longer durations. Here, we present empirical support for an alternative approach, the ‘Threat-of-Scream’ paradigm, in which shocks are replaced by screams. In two studies, participants were repeatedly exposed to blocks in which they were at risk of hearing aversive screams at any time vs. blocks in which they were safe from screams. Contrary to previous ‘Threat-of-Scream’ studies, we ensured that our screams were neither harmful nor intolerable by presenting them at low intensity. We found higher subjective reports of anxiety, higher skin conductance levels, and a positive correlation between the two measures, in threat compared to safe blocks. These results were reproducible and we found no significant change over time. The unpredictable delivery of low intensity screams could become an essential part of a psychology toolkit, particularly when investigating the impact of anxiety in a diversity of cognitive functions and populations.

## Introduction

Given the ubiquity and persistence of anxiety disorders, as well as their massive impact upon quality of life^1^, it is essential that neurobiologists and clinicians are able to determine how anxiety influences human brain physiology and behavioural responses to stress or external threats, along the continuum from normal to pathological conditions. While high anxiety leads to exaggerated estimates of the probability of threat, a certain level of anxiety is crucial for an organism’s survival as it ensures optimal sensitivity and decisiveness in the face of possible threat^2–4^.

The predictability of threat appears to be a major determinant of anxiety-related bodily manifestations, such as modulations of heart rate, startle reflex or skin conductance^5–8^. Indeed, predictable threats lead to phasic and acute fear responses (e.g. startle reflex^9^) that are directly associated with the appearance of that threat (e.g. a shock). In contrast, and in agreement with the safety-signal hypothesis^10^, unpredictable threats induce sustained anxiety-related physiological responses (e.g. startle reflex^9^; prepulse inhibition, a physiological marker of alertness^11^) and enhanced vigilance, reflected in a long-lasting facilitation of the processing of sensory information^12^.

The Threat of Shock paradigm (hereinafter TOS) has been the gold-standard paradigm to reveal the effects of anxiety on cognitive functions and quantify within-subject individual difference in threat response (for review^13^). TOS consists in alternating blocks in which participants are explicitly told that they could receive an electric shock at any time (unpredictable threat blocks) with blocks in which participants are explicitly told that no such shocks will occur (safe blocks). Such on–off alternations allow experimenters to manipulate the state of anxiety within subjects as each participant can serve as her/his own control. TOS proved to be a reliable method for inducing sustained anxiety, as reflected in participants’ higher physiological (startle reflex and elevated tonic skin conductance level) and psychological (higher reports of subjective anxiety) responses during threat versus safe blocks^8,14–16^.

Yet, similarly to all paradigms, TOS has limitations. First, it is not clear whether it can induce sustained anxiety responses for long durations. Experiments that have employed the TOS paradigm have been relatively short (~ 30 min). Hour-long experiments using TOS do exist^17^ but anxiety manipulation in these studies was mainly assessed using self-ratings of anxiety states, susceptible to demand effects, and/or local phasic physiological changes, which only represent a few seconds of the participants’ physiological state following the electric shock. To our knowledge, few experiments looked at whether induced anxiety could be maintained across several blocks. First, Bublatzky et al.^18^ reported no habituation of tonic skin conductance activity during an experiment lasting only 15 min, while Aylward et al.^19^ reported no habituation on subjective reports of anxiety during a 45-min experiment. Moreover, Bublatzky et al.^20^ provided promising results, even in the absence of aversive experiences, i.e. when participants believed that they could receive shocks in threat blocks while none were delivered, across repeated sessions within one day. However, in the absence of aversive stimulation, the anxiety induced across repeated test days diminished with different timing between subjective reports and physiological measures. It therefore remains unclear whether TOS, using unpredictable aversive stimulation, is resistant to physiological habituation. Yet, long-duration experiments are necessary when testing several conditions and/or requiring a large number of trials (as in computational modelling).

A second limitation has to do with the appropriateness of TOS for certain study populations. Although well-known for their aversive properties^21^, electric shocks may not be administrated to vulnerable and younger populations (notably children). White noise burst has been proposed as an alternative to electric shocks for fear-conditioning protocols that require a large number of trials (e.g. EEG and MEG, see^22^). However, the same criticism arises. Aversive noise bursts are often presented at between 95 and 110 dB, an intensity that can potentially be intolerable and which could cause hearing loss following prolonged exposition (European Legislation-directive n° 2003/10/CE). Sounds between 95 and 110 dB not only exceed tolerance (75 dB) and danger (85 dB) thresholds for audition, they come relatively close to the pain threshold of 120 dB.

What methodology can overcome these limitations while generating anxiety in most populations in a sustained and stable way? Threatening stimuli are useful insofar as they are perceived as unpleasant without being painful. In particular, distress screams produced by humans are good candidates as they are evolutionarily and socially meaningful sounds that efficiently signal impending danger to conspecifics^23,24^. They are perceived as highly aversive signals and are characterized by distinctive roughness acoustical properties^25^, which specifically engage subcortical regions known to be critical for swift reactions to danger^24^.

Human screams have previously been used during fear conditioning paradigms (e.g. screaming lady paradigm^26,27^), to investigate, for instance, the mechanisms involved in developmental anxiety^28^. Stimuli paired with screams induce higher skin conductance^29^ and startle responses^30,31^ as well as higher subjective anxiety reports^32^ compared to unpaired stimuli. However, as most past studies using screams delivered them with a potentially painful intensity (around or above 90 dB)^26,27,29,33–35^, the aversive nature of screams and the potential painful experience may have both contributed to the observed responses. Nevertheless, some studies did succeed in evoking acute fear responses using intensities lower or equal to 80 dB^30,32,36^. Of interest, the fear potential startle was found to be comparable for stimuli conditioned with electric shocks or 80 dB screams, even though those conditioned with shocks were reported to be more aversive than those conditioned with screams^30^. If the substitution of shocks by screams is a promising means of generating acute stress, the question remains as to whether human screams can be an efficient tool to induce sustained anxiety in a within-subject paradigm. Of interest, adolescents show elevated startle responses in blocks during which a fearful female face accompanied by a piercing loud scream was presented in an unpredictable manner (as compared to blocks with neutral cues), suggesting that scream stimuli can induce sustained state of anxiety^37^. Moreover, Patel et al.^38^ manipulated anxiety in adolescents performing a working memory task using loud shrieking screams (Threat of Scream paradigm—TOSc). Substituting shocks with screams was successful as participants reported being more anxious and had higher startle responses in threat blocks compared to safe ones. Subjective anxiety of participants was found to be constant while no information was provided about differences in physiological changes over time. Yet, these experiments were short-lasting and the screams were delivered at a high intensity (95-dB).

**Figure 1:**
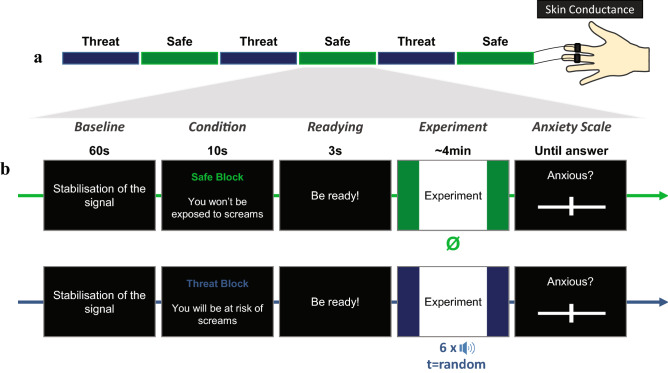
The Threat of Scream Design. (**a**) Temporal organisation of the experiment. Participants performed a free action-task, in the two types of alternating blocks (Safe/Threat). (**b**) Temporal organisation of a block. Each block began with one minute of baseline to measure participants’ skin conductance before each block. A written sentence was then presented for 10 s, providing information about the upcoming nature of the block (Safe or Threat). Each block ended with an anxiety scale. Note that skin conductance activity was measured throughout each block whereas subjective anxiety ratings were only collected at the end of each block.

To further establish the viability of the Threat of Scream paradigm (TOSc) and its promise for research on anxiety, we used human distress screams delivered at low intensity—rather than high intensity screams or electric shocks—to test their efficiency in inducing anxiety during a one-hour experiment (See Fig. 1). Since distress screams have specific acoustic properties and privileged communicative function, we expected these to be particularly suitable to evoke anxiety in a long-lasting fashion, especially when presented in an unpredictable manner. To determine whether sustained anxiety was induced, we measured subjective reports of anxiety and skin conductance activity; two markers that track environmental uncertainty^39^. We ran the same one-hour experiment twice, to assess whether the observed effects were replicable. Two conditions had to be met to validate the TOSc paradigm: (i) the unpredictable screams presented at low intensity (< 80 dB) should modulate anxiety responses, with increased subjective anxiety reports and increased tonic physiological activity (skin conductance) in threat compared to safe blocks, similarly to previous TOS studies; and (ii) sustained state anxiety should be induced for extended periods (here 1 hour).

## Results study 1

### Skin conductance level (SCL)

**Figure 2:**
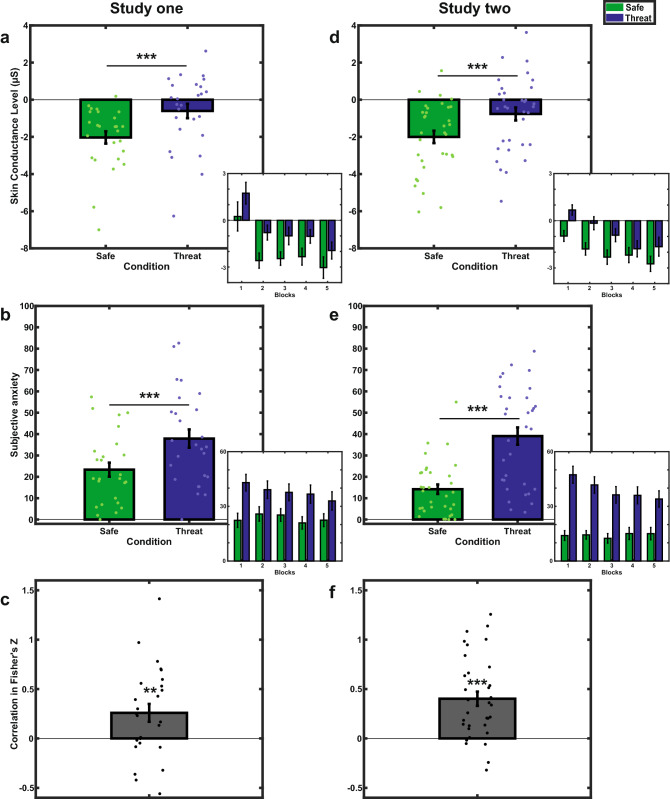
Skin conductance level (top), subjective anxiety (middle) and intra-individual correlation between SCL and subjective anxiety (bottom) for study one (left) and its replication study two (right). Error bars and points represent respectively standard errors and individual data. Miniatures provide descriptive variation of the reported effects relative to each block. ***p < 0.001; **p < 0.01; *p < 0.05; n.s. = p > 0.05.

Participants’ tonic skin conductance activity (SCL) was greater during Threat (M = − 0.61, SE = 0.39) relative to Safe (M = − 2.03, SE = 0.33) blocks (F(1,25) = 17.20, p < 0.001, ƞ^2^_p_ = 0.41) (Fig. 2a). We observed a main effect of Time (F(4,100) = 12.96, p_corr_ < 0.001, GG-ε = 0.49, ƞ^2^_p_ = 0.34), suggesting a decrease of SCL across the experiment. However, there was no significant interaction between Condition and Time (F(4, 100) = 0.12, p_corr_ = 0.92, GG-ε = 0.58, ƞ^2^_p_ = 0.005), suggesting that the difference in SCL between Safe and Threat did not change with time.

### Subjective reports of anxiety

Participants reported higher scores on the anxiety scale at the end of Threat (M = 37.90, SE = 4.27) compared to Safe (M = 23.35, SE = 3.28) blocks (F(1,25) = 15.11, p < 0.001, ƞ^2^_p_ = 0.38) (Fig. 2b). While factor Time was not significant (F(4,100) = 1.84, p_corr_ = 0.16, GG-ε = 0.61, ƞ^2^_p_ = 0.069), there was a trend toward significance for the interaction term between Condition and Time on subjective reports (F(4,100) = 2.14, p = 0.081, ƞ^2^_p_ = 0.079). Based on complementary t-tests (see Table S14), the interaction appears to be driven by a diminution of the difference in subjective anxiety between Threat and Safe conditions in the fifth and last block (for Blocks 1 to 4, all ps < 0.005 and Cohen’s d > 0.63; for Block 5, p = 0.032 and Cohen’s d = 0.45).

### Intra-individual correlation

The average of intra-individual correlation estimates between SCL and subjective reports was positive (Mean_r_ = 0.21, Mean_r-to-z_ = 0.26), of medium size and statistically different from zero (bidirectional t-test, t(25) = 2.86, p = 0.009, Cohen’s d = 0.56, lower 95% CI for Cohen’s d = 0.14, upper 95% CI for Cohen’s d = 0.97) (Fig. 2c).

### Inter-individual variability

In exploratory analyses, we assessed whether inter-individual variability in anxiety trait, measured by the STAI-trait auto-questionnaire, could explain part of the variance in our measures of interest (SCL, subjective reports of anxiety and intra-individual correlation). No significant interaction or correlation were observed with scores at the STAI-trait questionnaire (all the ps > 0.17, see Tables S17–S19).

## Results study 2 (replication)

### Skin conductance level (SCL)

Replicating results from Experiment 1 (Fig. 2d)**,** participants’ tonic skin conductance activity (SCL) was higher during Threat (M = − 0.77, SE = 0.35) relative to Safe (M = − 2.00, SE = 0.33) blocks (F(1,32) = 29.36, p < 0.001, ƞ^2^_p_ = 0.48). We observed a main effect of Time (F(4,128) = 12.48, p_corr_ < 0.001, GG-ε = 0.73, ƞ^2^_p_ = 0.28), suggesting a decrease of SCL across the experiment. However, there was no significant interaction between Condition and Time (F(4,128) = 1.35, p = 0.25, ƞ^2^_p_ = 0.041).

### Subjective reports of anxiety

Replicating results from Experiment 1, participants reported higher scores on the anxiety scale at the end of Threat (M = 39.08, SE = 4.55) compared to Safe (M = 14.19, SE = 2.51) blocks (F(1,32) = 47.84, p < 0.001, ƞ^2^_p_ = 0.60) (Fig. 2e). There was a trend toward significance for the main effect of Time, suggesting that participants’ subjective reports decreased in intensity across the experiment (F(4,128) = 2.47, p_corr_ = 0.083, GG-ε = 0.58, ƞ^2^_p_ = 0.072). There was a significant interaction between Condition and Time on subjective reports (F(4,128) = 4.05, p = 0.004, ƞ^2^_p_ = 0.11). Post-hoc tests revealed a significant decrease in the difference in subjective anxiety between Threat and Safe conditions between the first and the fourth and fifth blocks (B1vsB4: Mean Difference = 12.36, SE = 3.63, t(32) = 3.41, p_bonf_ = 0.018; B1vsB5: Mean Difference = 14.30, SE = 4.20, t(32) = 3.40, p_bonf_ = 0.018; all other comparisons ps > 0.342, see Table S29). Note however that the subjective difference between Threat and Safe conditions remained significant in all blocks (all the p < 0.001, 0.77 < Cohen’s d < 1.24) (See Table S31).

### Intra-individual correlation

Replicating results from Experiment 1, mean of intra-individual correlation between SCL and subjective reports of anxiety was positive (Mean_r_ = 0.33, Mean_r-to-z_ = 0.40), of large size and statistically different from zero (Bidirectional T-test-t(32) = 5.58, p < 0.001, Cohen’s d = 0.97, lower 95% CI for Cohen’s d = 0.55 upper 95% CI for Cohen’s d = 1.38) (Fig. 2f).

### Inter-individual variability

As in Study 1, we assessed whether inter-individual variability in trait anxiety, measured by the STAI trait auto-questionnaire, could explain part of the variance in our measures of interest (SCL, Subjective anxiety and intra-individual correlation). No significant interaction or correlation were observed with the scores in the STAI trait questionnaire (all the ps > 0.091, see Table S34–S36).

## Discussion

The present experiment aimed at offering additional validation to the Threat-of-Scream paradigm (TOSc^38^) by investigating whether sustained anxiety could be induced during a period of one hour, using unpredictable distress screams delivered at low sensory intensity (70-dB). We measured two proxies of anxiety, namely subjective reports of anxiety and skin conductance level, and ran the same hour-long experiment twice to assess the replicability of our results. Both experiments revealed higher skin conductance level (SCL) and self-reported anxiety during threat as compared to safe blocks. Moreover, we observed that the difference in SCL between safe and threat blocks remained constant throughout the experiment (or at least, statistically non-distinguishable), and that participants tended to report less anxiety towards the end of the experiment. The physiological state of participants (SCL) and their subjective reports of anxiety were positively correlated and all reported effect sizes were medium to large and replicable. Overall, our findings convincingly demonstrate the robustness of the TOSc paradigm as a tool to assess the relatively long-lasting impact of sustained anxiety with potential applications to a diversity of cognitive functions and populations.

To manipulate anxiety within participants, we used unpredictable human distress screams as aversive cues. Human distress screams are highly salient vocal signals of impending danger. Characterized by distinctive roughness acoustical properties which contribute to their aversiveness^24,40^, they are perceived as communicating fear^25^ and convey cues as to caller identity^41^. Past experiments successfully substituted shocks with human screams to generate acute stress during fear conditioning paradigms (e.g. screaming lady paradigm^26,27^). Comparable physiological responses (fear potential startle) were found for stimuli conditioned with electric shocks and 80 dB screams, even if stimuli conditioned with electric shocks were rated as more aversive than those conditioned with screams^30^.

Knowing that unpredictability can induce sustained anxiety provided that the anticipated stimulus is sufficiently aversive^9^, we tested whether unpredictable distress screams, delivered at lower sensory intensity (70-dB) than previous experiments^38,42^, could efficiently induce sustained anxiety. Our findings clearly demonstrate that the aversiveness of unpredictable distress screams at low sensory intensity (70-dB) is sufficient to induce sustained anxiety in a within-subject paradigm. Indeed, in addition to a modest but significant increase in self-reported anxiety, participants’ skin conductance level increased and a large difference in physiological activity (ƞ^2^_p_ > 0.40) was found in blocks during which human distress screams were delivered compared to safe blocks in both experiments.

Moreover, we demonstrate that anxiety can be induced for extended periods (here: one hour). We observed in two experiments that changes in tonic physiological activity (skin conductance level) between threat and safe blocks did not seem to be affected by time. Regarding self-reported anxiety, and contrary to Patel et al.^42^, we observed a decrease in subjective ratings of anxiety over time (significant in Study 2 and trending in Study 1), which could be explained by the duration of our experiment and/or by the intensity of the present screams. Yet, the difference in subjective anxiety between threat and safe conditions remained significant across all blocks. Our results complement previous findings of sustained tonic skin conductance activity during a 15-min study^18^ and of self-reported anxiety throughout a 30^42^ and 45-min experiment^19^. Altogether, findings from the current study indicate that unpredictable distress screams, which serve to communicate danger, (i) are efficient in manipulating anxiety within-subject for one hour, and (ii) can be perceived as aversive even at low intensity.

In the present experiment, screams were rare as they occurred in approximately 6% of trials (as in the original version developed by Patel et al.^38^). We isolated the tonic skin conductance activity by removing trials over which the exposure to screams could generate phasic activity. Moreover, the difference between threat and safe conditions can be observed by and was stable in the first seconds of the blocks, even when no scream had yet been heard (the first scream was delivered at 37 ± 4 s SD after the beginning of block, see Fig. S5). We therefore believe that our effect is related to the anxiety likely generated by the unpredictable threat context rather than by reactions to the screams themselves.

Subjective reports of anxiety and skin conductance level were found to be positively correlated in both experiments. The association between self-reported experience and observed physiological activity has been referred to as “emotion coherence”. It has been suggested that coherence across physiological, behavioural (facial expressions), and experiential responses is the definition of an emotion episode (e.g.^43–45^). This proposition is however debated, as some authors suggest that emotion systems are only loosely coupled^46,47^, or even independent (e.g.^48^). Results from past experiments in healthy subjects are inconclusive as they provided evidence either for a moderate association between physiological responses and self-rated experience^49–53^ or were consistent with the hypothesis that there is no emotional coherence between subjective and physiological data^54–56^.

Within the fear conditioning literature, positive correlations between SCR and self-reported experience have been observed^57,58^. For instance, Glenn, Lieberman, Hajcak^30^ observed, at a trend level, a convergence between subjective and physiological (fear-potentiated startle) measures of fear, but only for stimuli that were conditioned using electric shocks and not for those conditioned with screams. However, Abend et al.^59^ revealed a convergence of subjective and psychophysiological measures for CS + following conditioning, using 95 dB female scream. Here, by alternating blocks during which participants were at risk of hearing unpredictable aversive screams with blocks during which no screams were to be delivered (safe blocks), we show that unpredictable distress screams at low sensory intensity (70-dB) are sufficiently aversive to generate emotion coherence, i.e. positive correlation between subjective reports of anxiety and skin conductance level. Such coordinated changes (coherence) across physiological and experiential responses clearly support the efficiency of our manipulation in inducing an emotion (anxiety) episode.

Several limitations to this experiment need to be acknowledged. First, as in the majority of the threat of shock studies (i.e.^60,61^), aversive stimuli were delivered only in threat blocks while participants did not receive any stimulation in safe ones. This difference in stimulation could be involved in the observed SCL differences. Future studies should eliminate this confound. We envision three possibilities here. First, one could avoid stimulation entirely during the task. Indeed, some experiments have either delivered shocks during a shock work-up procedure before the main experiment^18,63^ or never delivered shocks to participants^62^. Subsequent verbal instructions (i.e., “you can be exposed to shocks in this block”) and cues that falsely signal the possibility of a shock were sufficient to elicit autonomic and subjective fear. A second possibility is to deliver the aversive stimuli at the end of the threat block to best isolate their effects^64^. However, participants appeared to learn that they were in fact safe of shocks in so-called threat blocks when the experiment was of a long duration^20^. A third possibility is to add monetary stimulation in safe blocks, which causes stimulation and makes these blocks ‘appetitive’ rather than neutral^14,65^. Nevertheless, these 3 methods still do not appear to be fully appropriate to specifically investigate long-lasting anxiety.

Second, one may question the specificity of tonic skin conductance activity (SCL) to assess sustained anxiety. SCL is known to be modulated by different factors such as participants’ emotional state, arousal, visual attention and motor activity^66, 67^. As such, SCL may only represent an indirect marker of anxiety (see^9^), and the present higher SCL during threat compared to safe blocks may be linked to one or more of these modulators, i.e. higher level of arousal, and/or negative emotional state and/or increase of visual attention. There is however some evidence in the literature that SCL can reflect participant’s sustained anxiety. For instance, Doberenz et al.^68^ revealed elevated SCL in awake panic disorder patients compared to controls, and suggested that it was related to sustained anxiety between panic attacks, i.e. anxious anticipation of future attacks. Recently, and in a more controlled setting, Neueder et al.^69^ show that successful context conditioning is indicated by higher anxiety ratings and skin conductance levels (SCLs) in an anxiety context where an aversive unconditioned stimulus occurred unpredictably vs. a safety context. Finally, De Becker et al.^39^ demonstrated that one physiological marker that tracks environmental uncertainty is skin conductance activity. Altogether, these findings indicate that SCL could capture participants’ anxiety induced by the unpredictable delivery of screams. Future studies should however confirm our results by measuring the startle reflex, one of the most common readouts in the context of predictable/unpredictable threat (e.g.^9,37^).

Third, sustained contextual anxiety, but not phasic fear to a predictable threat, is believed to differentiate anxious from non-anxious individuals (e.g.^70^). The present absence of association between our measures of interest, i.e. SCL and subjective report of anxiety, and participants’ trait anxiety could be seen as a challenge for the validity of our paradigm. A first potential explanation is that the relatively limited sample size did not provide sufficient power to reliably examine correlations among these measures. However, looking at previous studies that used the threat-of-shock or -scream paradigms to induce state anxiety, the association between participants’ trait anxiety and both subjective and physiological measures is unclear. Indeed, most studies in normative samples that we are aware of only report the mean trait anxiety scores of their participants without performing any correlations between trait anxiety and physiological measures (e.g.^9,64,71–73^, with the exception of^42^). Future experiments should therefore further examine the interactions between (shock- and scream-induced) state anxiety and temperamental trait anxiety disposition.

Fourth, there may be an influence of the action-decision task on our anxiety measures. This is indeed a possibility as (a) the present task consists in taking-action decisions in the presence of two task-irrelevant individuals, one of them displaying in 2/3 of the trials a threat-related expression (fear or anger); and (b) Grillon and Charney^71^ have revealed that the startle reflex was transiently potentiated by fearful faces compared to neutral faces in threat periods, suggesting that fearful faces can prompt behavioral mobilization in an anxiogenic context. The startle reflex is a reflexive reaction to an unexpected and intense stimulus, which is reliably potentiated by negative emotional states (e.g.^74^). According to this definition, phasic skin conductance response following unexpected and negative stimuli may be similarly impacted in threat contexts. Therefore, one could envisage higher phasic skin conductance activity to the screams and to the presence of a threat-related facial expression in threat as compared to safe blocks. However, as we filtered our data in order to reduce the phasic component of the skin conductance activity, we believe that such an effect cannot entirely explain the difference we observed in tonic skin conductance activity (SCL) between threat and safe blocks. Regarding subjective reports of anxiety, we cannot fully exclude the possibility of an influence of the ongoing task (and stimuli), as Grillon and Charney^71^ observed a significant positive correlation between the startle potentiation score (difference between fear and neutral stimuli) and the increased fear reported by the subjects during threat relative to safe contexts. Future studies are needed to confirm our results, by measuring the startle reflex to startle stimuli in both threat and safe contexts.

Finally, the present study does not demonstrate that the threat-of-scream paradigm leads to longer lasting effects than the threat-of-shock paradigm. However, as electric shocks, which represent a direct physical threat, may be considered more aversive than human screams (electric shocks constitute a threat; screams are signals of impending threat), the associated induced anxiety should be even less susceptible to time. Also, if the aversiveness of the delivered stimuli is one important component in the induction of anxiety, the unpredictability of the aversive stimuli is probably the main component in such paradigms. As both paradigms share these two major components, we are confident that future experiments will provide evidence for long-lasting effects using the threat-of-shock paradigm.

Overall, our study offers support to the TOSc paradigm by showing that: (i) unpredictable distress screams presented at low intensity (< 80 dB) can induce sustained anxiety as revealed by increased subjective reports of anxiety and increased tonic physiological activity (skin conductance level) during threat compared to safe blocks, similarly to previous threat-of-shock experiments; and that (ii) sustained states of anxiety can be induced for extended periods (here one hour). Distress screams, delivered at lower sensory intensity (70-dB), thus appear to be excellent candidates to overcome the ethical issues associated with exposing vulnerable and young populations to electric shocks and aversive noise, and to experimentally address the emergence of pathological anxiety in a consistent and sustained manner.

## Methods

### Participants

Twenty-six healthy volunteers (12 females, age 23.6 ± 3.4 years SD) were recruited to participate in Experiment 1 (a sample of the same magnitude as Patel et al. (2016)’s study). Results from the correlation between tonic skin conductance activity and subjective reports of anxiety were used to calculate the sample size needed to replicate this result using G*power. The sample size for replication was estimated at n = 27 for an effect size of d = 0.56, α = 0.05 and β = 0.80. To anticipate potential exclusions, we aimed at including 35 participants in Experiment 2 (Replication of Experiment 1), and 33 participants could be recruited (18 females, age = 23.89 ± 4.50 years SD).

All participants were right-handed, had normal or corrected-to-normal vision, and had no history of neurological or psychiatric disorders. The experimental protocol was approved by INSERM and the local research ethics committee (Comité de protection des personnes Ile de France III—Project CO7-28, N° Eudract: 207-A01125-48), and it was carried out in accordance with the Declaration of Helsinki. The participants provided informed written consent and were compensated for their participation.

### General procedure

First, participants were recruited thanks to an online advert, which consisted of a short description of the study and included an internet link which directed participants to a Qualtrics survey. Due to the potentially stressful nature of our paradigm and after discussion with the referent medical doctors of our laboratory, we requested that participants fill in, online and anonymously, the French versions of State-Trait Anxiety Inventory Spielberger^75^ (STAI) and Post-traumatic stress disorder Checklist Scale^76^ (PCLS), at least one week before the experiment. Only participants with a score below 40 for the PCLS and below 60 for both STAI state and trait anxiety were able to contact the experimenter, i.e. and were therefore included in the experiment.

Second, and on the day of the experiment, upon arrival at the lab, participants again completed the State-Trait STAI questionnaire on a computer to ensure that their level of anxiety was in the “normal” range (below 60) before the experimental session that aimed at manipulating their anxiety (see Figs. S1, S2).

Third, after providing the instructions regarding the main task, the experimenter installed the skin conductance electrodes. To identify potential physiological non-responders before the main experiment, the experimenter assessed the variation of the physiological signal while participants were asked to imagine a situation during which they fell into really cold water, and this, until stopped by the experimenter. All our participants showed increased physiological response during this mental imagery task; the response then dropped when asked to stop the exercise. Based on this definition of ‘non-responder’, there were no non-responders among our participants.

Fourth, participants started with a 4-min training session, before performing the main experiment for approximately one hour, and were carefully debriefed at the end of the main experiment.

### Screams stimuli

Eight distress screams were used in the present protocol (4 from males and 4 from females). The screams were normalized at -2b using audiosculpt 3.4.5 (https://forumnet.ircam.fr/shop/fr/forumnet/10-audiosculpt.html). During both the training session and the main experiment, screams were delivered using Bose headphones (QuietComfort 25) at peak intensity below 70 dB (mean of 68 dB as measured by a sonometer).

The screams were provided by Professor Armony, and had been previously validated^77^ by a group of 60 individuals who rated their emotional valence and intensity. However, at the end of the experiment, during the debriefing, we collected ratings for all screams together on an aversive scale (from 0—not at all aversive to 10—extremely aversive). Participants rated the screams as moderately aversive (median of 4, see Fig. S4, left). Note however that, compared to past studies, we delivered the screams at a lower intensity (70 dB rather than 85–90 dB as in^78^). As inducing anxiety relies both on the aversiveness of the delivered stimuli as well as their unpredictability, we also asked participants to rate how much they felt preoccupied by the possibility that a scream would be delivered during threat blocks (this was done during the debriefing). Participants felt relatively preoccupied, i.e. they reported anticipatory anxiety of scream delivery (median of 6 see Fig. S4, right). They often stated that they were wondering when the next scream was to appear.

### Experimental design and task

The experiment consisted in an alternation between threat and safe blocks (Fig. 1a). Participants were informed that during threat blocks, the sides of the screen were blue, meaning that they were at risk of hearing unpredictable distress screams at any time, through their headphones. In contrast, during safe blocks, the sides of the screen were green meaning no screams were to be delivered.

During both threat and safe blocks, participants performed a free action-decision task in a social context, developed by Vilarem et al.^79^. Each trial started with a grey screen of 1,000 ms, followed by a fixation cross for 500 ms. A picture of a scene was then presented which remained on the screen until a response was registered, or until a maximum time of 1,400 ms in the case of no response (each trial has therefore a duration of 2,900 ms maximum). The scene depicted a waiting room with four chairs, of which the outer two were empty. The two middle chairs were occupied by two task-irrelevant individuals, one displaying a neutral expression while the other displayed either a neutral, fearful or angry expression. Participants were asked to indicate the seat they would like to occupy, maintaining fixation on the cross displayed between the faces throughout the trial. In order to make their choice, participants had to left-click on the mouse, move the cursor from the bottom center of the scene and release the click on the chosen seat. The cursor was automatically re-centered at every new trial. Participants were required to make spontaneous choices and were informed that there were no correct or incorrect responses. Importantly here, the number of neutral, fear and anger trials was equal for safe and threat blocks. Data from the task are not presented here, being part of another study (Beaurenaut et al. unpublished). To resume, participants performed a free action-task and were either exposed to screams at any time during threat blocks and to no screams during safe blocks.

The temporal organisation of each block was as follows (Fig. 1b): a black screen with the written instruction “stabilisation of the signal” was first presented for 1 min during which the physiological baseline of participants was recorded. Second, to inform participants about the nature of the block, the written instruction “Threat Block: at any time, a scream can be presented” or “Safe Block: you will hear nothing during this block” was presented for 10 s. Third, participants performed the above-described free action-task for about 4 min. Fourth, at the end of each block, participants reported their anxiety level on a continuous scale (from 0: really calm to 100: really anxious), by moving a cursor on the screen using the mouse. The scale was presented on the computer screen and disappeared once the response was given. Finally, to assess physiological variation of participants’ bodily state in threat compared to safe blocks, skin conductance activity was recorded throughout the blocks.

A training session was performed before the main experiment to familiarize participants with the free action-task, the structure of the experiment (the alternation of safe and threat blocks and their associated screen sides colours) and the screams. Participants were trained on 2 blocks, one safe and one threat, each of 32 trials. During the threat block, 2 distress screams (one male and one female) were delivered once during two randomly chosen trials from among the 32 (approximately 6% of the trials). Note that the two screams used for the training were different from the 6 screams used during the main experimental.

Participants then performed the main experiment (M_duration_ = 65 min, SD_duration_ = 5 min), divided into 10 blocks of 96 trials, 5 threat blocks and 5 safe blocks. During each threat block, 6 distress screams were delivered once, randomly during the block (approximately 6% of the trials), either before the grey screen (2 screams), before the fixation cross (2 screams), or at the appearance of the scene (2 screams). The nature (safe or threat) of the first block was counterbalanced across participants: the threat blocks were the blocks 2–4–6–8–10 for even participants and 1–3–5–7–9 for the odd participants.

### Skin conductance recordings

Skin conductance activity was recorded using a PowerLab 8/35 amplifier, with a GSR Amp (FE116) unit which uses low, constant-voltage AC excitation (22 mV rms at 75 Hz), and a pair of stainless steel dry bipolar electrodes (3 × 2.5 cm, MLT118F). The electrodes were attached to the participant's left index and ring fingers of the non-dominant hand, using dedicated Velcro. Recordings were performed with LabChart 7 software, at a sampling rate of 1 kHz, with the recording range set to 40 μS and using initial baseline correction (“subject zeroing”) to subtract the participant's absolute level of electrodermal activity from all recordings (devices and software from ADInstruments). Finally, as low-pass filter of 0.05 Hz has been proposed as an appropriate filter to eliminate any noise as well as the phasic component of the signal (see^80^), we applied this filter to isolate the tonic activity of the skin conductance signal and to minimize the phasic influence of screams during threat blocks on the skin conductance activity. Performing the analyses on raw data signal revealed similar results as those performed on filtered data (see Fig. S3).

### Skin conductance level (SCL) processing

The SCL corresponds to the tonic activity of the skin conductance signal. The physiological signal was processed using Labchart 7 and Matlab. For each participant and for each block, we obtained (a) one SCL value corresponding to the averaged signal over the 1-min baseline at the start of the block (see Fig. 1b), and (b) one SCL value corresponding to the averaged signal over task performance (M_Duration of one block_ = 4 min, SD = 30 s). The instruction phase (Condition + Readying—see Fig. 1b), between 1-min baseline and the beginning of the free action-task was not included in the averaged signal. Moreover, for threat blocks only, the phasic activity induced by the distress screams (6% of threat block trials) was excluded from the averaged signal. Finally, for each block (whether safe or threat), variation from the baseline was obtained by subtracting the baseline activity from the averaged activity over task performance. We therefore obtained, for each participant, 5 values corresponding to the 5 Safe blocks, and 5 values corresponding to the 5 Threat blocks.

### Statistical analyses

All statistical analyses were carried out using JASP Software (JASP Team (2017), JASP (Version 0.8.5.1) [Computer software]). The corresponding tables of results are available in Supplementary materials.

#### Repeated-measures ANOVAs

For each experiment and for both the physiological variable (SCL) and the subjective reports of anxiety, we ran two-way repeated-measures ANOVAs with Condition (Threat vs. Safe) and Time (Blocks 1 to 5) as within-subject factor. We applied the Greenhouse–Geisser correction to correct for deviations from the assumption of sphericity (the corrected *P corrected* and the GG-ε are reported) and Bonferroni correction for the post-hoc tests (P*bonf*). Effect sizes (partial eta-squared, η^2^_p_) are reported together with F and p values. To better examine if SCL and subjective anxiety changed over time, we looked at threat vs. safe difference over time (see Supplementary Materials).

#### Intra-individual correlation

To assess the intra-individual coherence between the physiological state of participants and their subjective experience, we first computed Pearson’s r correlation coefficient for each participant between their subjective reports and SCL measures (10 values for each measure and each participant, since the experiment was composed of 10 blocks). We then performed Fisher’s r‐to‐z transformation to normalize Pearson’s r correlation coefficients^81^ before testing whether the correlation coefficients across participants were different from zero (bidirectional one-sample t-test).

#### Inter-individual variability

We assessed whether inter-individual variability in trait anxiety could partly explain the variance in our measures of interest (SCL, subjective anxiety, and intra-individual correlation). To do so, we re-ran the above-described ANOVAs for SCL and subjective anxiety with the scores of the STAI-trait questionnaire as co-variable, and performed a correlation between the intra-individual correlations (used to assess emotion coherence) and the scores of the STAI-trait questionnaire.

## Supplementary information

Supplementary Information.

## Data Availability

Data and analysis are available on OSF. link: https://osf.io/f2bvr/?view_only=4a4fda675e8d41f7bf20a9033f013a6f

## References

[1] Leon AC, Portera L, Weissman MM (1995). The social costs of anxiety disorders. Br. J. Psychiatry.

[2] Bateson M, Brilot B, Nettle D (2011). Anxiety: an evolutionary approach. Can. J. Psychiatry.

[3] Grillon C (2008). Models and mechanisms of anxiety: evidence from startle studies. Psychopharmacology.

[4] Marks IFM, Nesse RM (1994). Fear and fitness: an evolutionary analysis of anxiety disorders. Ethol. Sociobiol..

[5] Alvarez RP, Chen G, Bodurka J, Kaplan R, Grillon C (2011). Phasic and sustained fear in humans elicits distinct patterns of brain activity. Neuroimage.

[6] Vansteenwegen D, Iberico C, Vervliet B, Marescau V, Hermans D (2008). Contextual fear induced by unpredictability in a human fear conditioning preparation is related to the chronic expectation of a threatening US. Biol. Psychol..

[7] Davis M, Walker DL, Miles L, Grillon C (2010). Phasic vs sustained fear in rats and humans: Role of the extended amygdala in fear vs anxiety. Neuropsychopharmacology.

[8] Torrisi S (2016). The neural basis of improved cognitive performance by threat of shock. Soc. Cogn. Affect. Neurosci..

[9] Grillon C, Baas JP, Lissek S, Smith K, Milstein J (2004). Anxious responses to predictable and unpredictable aversive events. Behav. Neurosci..

[10] Seligman MEP, Binik YM (1977). The safety signal hypothesis. Operant. Interact..

[11] Grillon C, Davis M (1997). Effects of stress and shock anticipation on prepulse inhibition of the startle reflex. Psychophysiology.

[12] Kastner-Dorn AK, Andreatta M, Pauli P, Wieser MJ (2018). Hypervigilance during anxiety and selective attention during fear: using steady-state visual evoked potentials (ssVEPs) to disentangle attention mechanisms during predictable and unpredictable threat. Cortex.

[13] Robinson OJ, Vytal K, Cornwell BR, Grillon C (2013). The impact of anxiety upon cognition: perspectives from human threat of shock studies. Front. Hum. Neurosci..

[14] Bradley MM, Zlatar ZZ, Lang PJ (2018). Startle reflex modulation during threat of shock and “threat” of reward. Psychophysiology.

[15] Grillon C, Robinson OJ, Mathur A, Ernst M (2016). Effect of attention control on sustained attention during induced anxiety. Cogn. Emot..

[16] Hubbard CS (2011). Modulation of nociceptive and acoustic startle responses to an unpredictable threat in men and women. Pain.

[17] Engelmann JB, Meyer F, Fehr E, Ruff CC (2015). Anticipatory anxiety disrupts neural valuation during risky choice. J. Neurosci..

[18] Bublatzky F, Guerra PM, Pastor MC, Schupp HT, Vila J (2013). Additive effects of threat-of-shock and picture valence on startle reflex modulation. PLoS ONE.

[19] Aylward J (2019). Altered learning under uncertainty in unmedicated mood and anxiety disorders. Nat. Hum. Behav..

[20] Bublatzky F, Gerdes ABM, Alpers GW (2014). The persistence of socially instructed threat: two threat-of-shock studies. Psychophysiology.

[21] Schmitz A, Grillon C (2012). Assessing fear and anxiety in humans using the threat of predictable and unpredictable aversive events (the NPU-threat test). Nat. Protoc..

[22] Sperl MFJ, Panitz C, Hermann C, Mueller EM (2016). A pragmatic comparison of noise burst and electric shock unconditioned stimuli for fear conditioning research with many trials. Psychophysiology.

[23] Belin P, Zatorre RJ (2015). Neurobiology: sounding the alarm. Curr. Biol..

[24] Arnal LH, Flinker A, Kleinschmidt A, Giraud AL, Poeppel D (2015). Human screams occupy a privileged niche in the communication soundscape. Curr. Biol..

[25] Anikin A, Bååth R, Persson T (2018). Human non-linguistic vocal repertoire: call types and their meaning. J. Nonverb. Behav..

[26] Lau JYF (2008). Fear conditioning in adolescents with anxiety disorders: results from a novel experimental paradigm. J. Am. Acad. Child Adolesc. Psychiatry.

[27] Lau JY (2011). Distinct neural signatures of threat learning in adolescents and adults. Proc. Natl. Acad. Sci..

[28] Britton JC, Lissek S, Grillon C, Norcross MA, Pine DS (2011). Development of anxiety: the role of threat appraisal and fear learning. Depress. Anxiety.

[29] Ahrens LM (2016). Fear conditioning and stimulus generalization in patients with social anxiety disorder. J. Anxiety Disord..

[30] Glenn CR, Lieberman L, Hajcak G (2012). Comparing electric shock and a fearful screaming face as unconditioned stimuli for fear learning. Int. J. Psychophysiol..

[31] Haddad ADM, Xu M, Raeder S, Lau JYF (2013). Measuring the role of conditioning and stimulus generalisation in common fears and worries. Cogn. Emot..

[32] Den ML, Graham BM, Newall C, Richardson R (2015). Teens that fear screams: A comparison of fear conditioning, extinction, and reinstatement in adolescents and adults. Dev. Psychobiol..

[33] Hamm AO, Vaitl D, Lang PJ (1989). Fear conditioning, meaning, and belongingness: a selective association analysis. J. Abnorm. Psychol..

[34] Dibbets P, Evers EAT (2017). The influence of state anxiety on fear discrimination and extinction in females. Front. Psychol..

[35] Geller DA (2017). Fear conditioning and extinction in pediatric obsessive compulsive disorder. Ann. Clin. Psychiatry.

[36] Glenn CR (2012). The development of fear learning and generalization in 8–13 year-olds. Dev. Psychobiol..

[37] Schmitz A (2011). Measuring anxious responses to predictable and unpredictable threat in children and adolescents. J. Exp. Child Psychol..

[38] Patel N (2016). Interaction of threat and verbal working memory in adolescents. Psychophysiology.

[39] De Berker AO (2016). Computations of uncertainty mediate acute stress responses in humans. Nat. Commun..

[40] Arnal LH, Kleinschmidt A, Spinelli L, Giraud A-L, Mégevand P (2019). The rough sound of salience enhances aversion through neural synchronisation. Nat. Commun..

[41] Engelberg JWM, Schwartz JW, Gouzoules H (2019). Do human screams permit individual recognition?. PeerJ.

[42] Patel N, Stoodley C, Pine DS, Grillon C, Ernst M (2017). Interaction of induced anxiety and verbal working memory: influence of trait anxiety. Learn. Mem..

[43] Tomkins SS (1962). Affect Imagery Consciousness: Volume I: The Positive Affects.

[44] Panksepp, J. The basics of basic emotion. *Nat. Emot. Fundam. Quest.* 20–24 (1994).

[45] Ekman P (1993). Facial expression and emotion. Am. Psychol..

[46] Bonanno G, Keltner D (2004). Brief report: the coherence of emotion systems: comparing “on-line” measures of appraisal and facial expressions, and self-report. Cogn. Emot..

[47] Izard, C. E. Differential emotions theory. in *Human emotions* 43–66 (Springer, New York, 1977).

[48] LeDoux JE, Pine DS (2016). Using neuroscience to help understand fear and anxiety: a two-system framework. Am. J. Psychiatry..

[49] Cuthbert BN, Schupp HT, Bradley MM, Birbaumer N, Lang PJ (2000). Brain potentials in affective picture processing: covariation with autonomic arousal and affective report. Biol. Psychol..

[50] Mauss IB, Levenson RW, McCarter L, Wilhelm FH, Gross JJ (2005). The tie that binds? Coherence among emotion experience, behavior, and physiology. Emotion.

[51] Nandrino J-L (2012). Cognitive and physiological dissociations in response to emotional pictures in patients with anorexia. J. Psychosom. Res..

[52] Franklin TB (2017). Prefrontal cortical control of a brainstem social behavior circuit. Nat. Neurosci..

[53] Dan-Glauser ES, Gross JJ (2013). Emotion regulation and emotion coherence: evidence for strategy-specific effects. Emotion.

[54] Vuilleumier P, Armony JL, Driver J, Dolan RJ (2001). Effects of attention and emotion on face processing in the human brain: an event-related fMRI study. Neuron.

[55] Vuilleumier P, Pourtois G (2007). Distributed and interactive brain mechanisms during emotion face perception: evidence from functional neuroimaging. Neuropsychologia.

[56] Morris JS, DeGelder B, Weiskrantz L, Dolan RJ (2001). Differential extrageniculostriate and amygdala responses to presentation of emotional faces in a cortically blind field. Brain.

[57] Lovibond PF, Davis NR, O’Flaherty AS (2000). Protection from extinction in human fear conditioning. Behav. Res. Ther..

[58] Rodriguez BI, Craske MG, Mineka S, Hladek D (1999). Context-specificity of relapse: effects of therapist and environmental context on return of fear. Behav. Res. Ther..

[59] Abend, R. *et al.* Anticipatory threat responding: associations with anxiety, development, and brain structure. *Biol. Psychiatry* (2019). 10.1016/j.biopsych.2019.11.006 PMC7211142 31955915

[60] Balderston NL, Hsiung A, Ernst M, Grillon C (2017). Effect of threat on right dlPFC activity during behavioral pattern separation. J. Neurosci..

[61] Aylward J (2017). The impact of induced anxiety on affective response inhibition. R. Soc. Open Sci..

[62] Costa VD, Bradley MM, Lang PJ (2015). From threat to safety: instructed reversal of defensive reactions. Psychophysiology.

[63] Bublatzky F, Schupp HT (2012). Pictures cueing threat: brain dynamics in viewing explicitly instructed danger cues. Soc. Cogn. Affect. Neurosci..

[64] Robinson OJ, Letkiewicz AM, Overstreet C, Ernst M, Grillon C (2011). The effect of induced anxiety on cognition: threat of shock enhances aversive processing in healthy individuals. Cogn. Affect. Behav. Neurosci..

[65] Cohen AO (2016). When is an adolescent an adult? Assessing cognitive control in emotional and nonemotional contexts. Psychol. Sci..

[66] Boucsein W (2012). Electrodermal activity.

[67] Critchley HD (2002). Electrodermal responses: what happens in the brain. Neurosci..

[68] Doberenz S, Roth WT, Wollburg E, Breuninger C, Kim S (2010). Twenty-four hour skin conductance in panic disorder. J. Psychiatr. Res..

[69] Neueder D, Andreatta M, Pauli P (2019). Contextual fear conditioning and fear generalization in panic disorder. Front. Behav. Neurosci..

[70] Grillon C, Morgan CA, Davis M, Southwick SM (1998). Effects of experimental context and explicit threat cues on acoustic startle in Vietnam veterans with posttraumatic stress disorder. Biol. Psychiatry.

[71] Grillon C, Charney DR (2011). In the face of fear: anxiety sensitizes defensive responses to fearful faces. Psychophysiology.

[72] Grillon C (2020). Better cognitive efficiency is associated with increased experimental anxiety. Psychophysiology.

[73] Sarigiannidis I, Grillon C, Ernst M, Roiser JP, Robinson OJ (2020). Anxiety makes time pass quicker while fear has no effect. Cognition.

[74] Lang PJ, Bradley MM, Cuthbert BN (1990). Emotion, attention, and the startle reflex. Psychol. Rev..

[75] Spielberger, C. D. State-trait anxiety inventory for adults. (1983).

[76] Weathers, F. W., Litz, B. T., Herman, D. S., Huska, J. A. & Keane, T. M. The PTSD Checklist (PCL): Reliability, validity, and diagnostic utility. in *Annual Convention of the International Society for Traumatic Stress Studies, San Antonio, TX* **462**, (San Antonio, TX, 1993).

[77] Fecteau S, Armony JL, Joanette Y, Belin P (2005). Judgment of emotional nonlinguistic vocalizations: age-related differences. Appl. Neuropsychol..

[78] Fecteau S, Belin P, Joanette Y, Armony JL (2007). Amygdala responses to nonlinguistic emotional vocalizations. Neuroimage.

[79] Vilarem, E., Armony, J. L. & Grèzes, J. Action opportunities modulate attention allocation under social threat. *Emotion* (2019). 10.1037/emo0000598 30945887

[80] Braithwaite JJ, Watson DG, Jones R, Rowe M (2013). A guide for analysing electrodermal activity (EDA) & skin conductance responses (SCRs) for psychological experiments. Psychophysiology.

[81] Howell DC (2009). Statistical Methods for Psychology.

