# Supplementary material for: The ‘Threat of Scream’ paradigm: a tool for studying sustained physiological and subjective anxiety
Source: Sci Rep. 2020 Jul 27;10:12496. doi: 10.1038/s41598-020-68889-0 (PMC7385655; doi:10.1038/s41598-020-68889-0)
Supplement: Supplementary file 1 — Supplementary Information. [file 41598_2020_68889_MOESM1_ESM.docx]

# Supplementary Material

**The ‘Threat of Scream’ paradigm: A tool for studying sustained physiological and subjective anxiety**

Morgan Beaurenaut^a*^, Elliot Tokarski ^a^, Guillaume Dezecache^b,c^^ & Julie Grèzes^a^^*

^a^Laboratoire de Neurosciences Cognitives et Computationnelle, Département d’études cognitives, ENS, PSL Research University, INSERM, Paris France

^b^Department of Experimental Psychology, Division of Psychology and Language Sciences, University College London, London, United Kingdom

^c^Université Clermont Auvergne, CNRS, LAPSCO, Clermont-Ferrand, France

**^^^**Equal contribution

*****Authors for correspondence: MB (beaurenaut.morgan@gmail.com) and JG (julie.grezes@ens.fr)

# Description

The present work aimed at assessing the efficiency of a new version of TOSc paradigm during which we delivered unpredictable human distress screams at low intensity (70dB instead of 95dB used in the past). In the main manuscript, we discussed findings related to tonic skin conductance activity (which represent the physiological activity of participant through the entire block) and subjective scores of anxiety reported by participants at the end of each block. This supplementary document provides detailed about the Methods (the characteristics of the sampling distribution, debriefing that concluded the experimental session, signal quality check) as well as the tabs of results for all the analyses reported in the main text (Statistics, Repeated Measures ANOVA, Post Hoc Comparisons and One Sample T-Test Against Zero).

# Methods – Supplementary information

### Participants

We provide the mean and SD, as well as the distribution of scores to STAI state and trait questionnaires, for each experiment.

| **Table S1.** Characteristics of the sample of Study one | | | | |
| --- | --- | --- | --- | --- |
| **Size** | **Gender** | **Age** | **STAI-State** | **STAI-Trait** |
| 26 | 12 F/14 M | 23.6 ± 3.4 y.o SD | 28.9 ± 6.1 SD | 37.4 ± 7.2 SD |


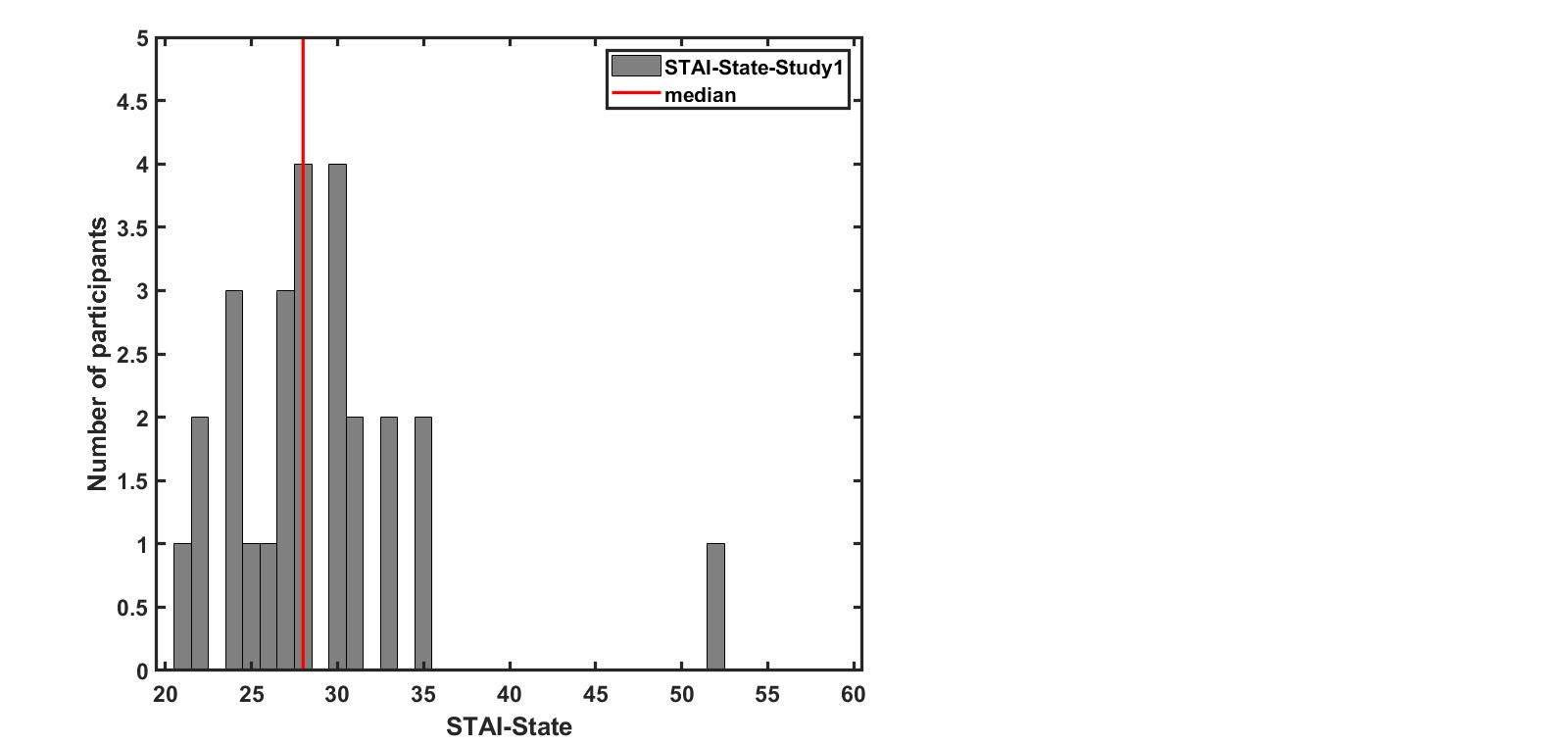

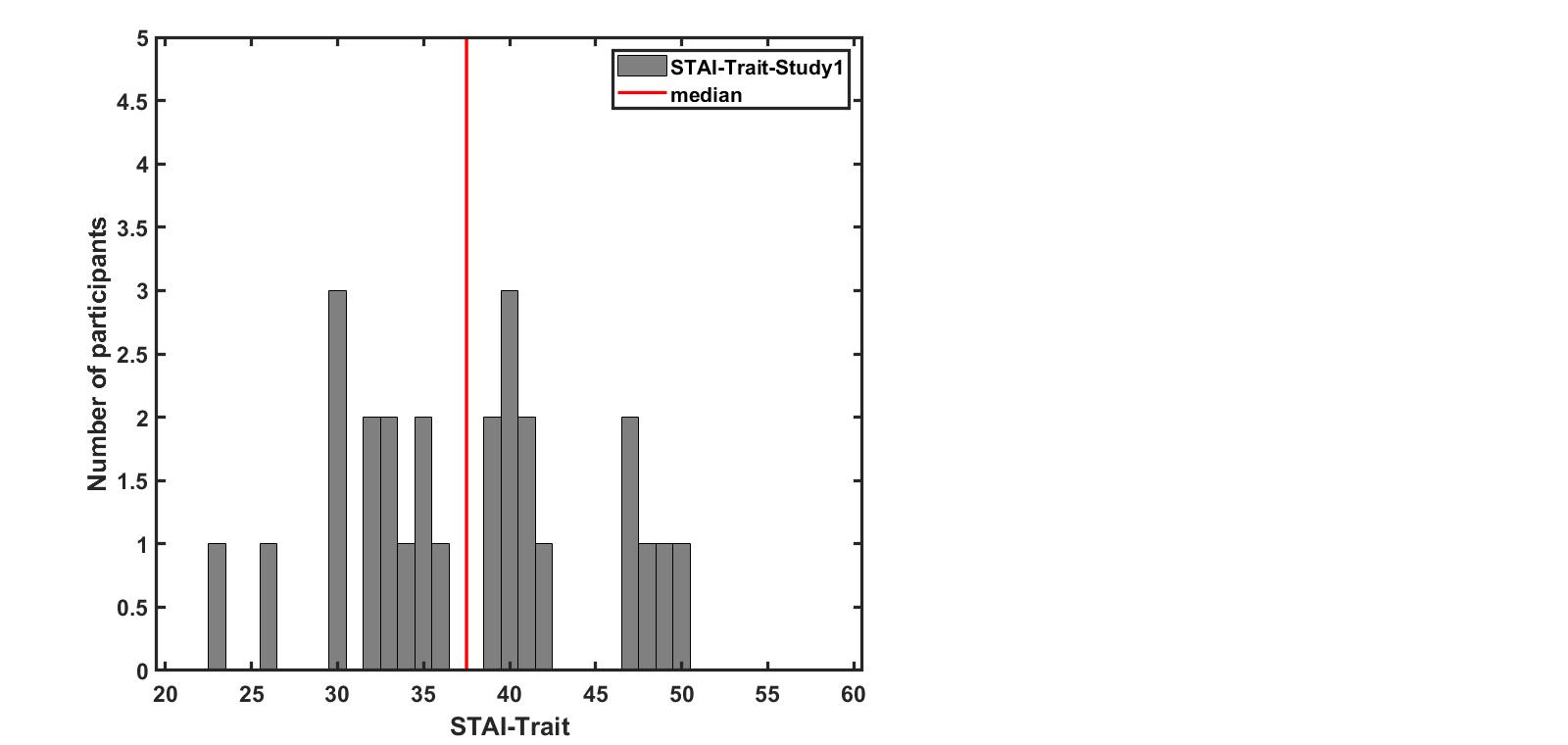


**Figure S1.** Distribution of STAI-State (Left) and STAI-Trait (Right) of Study one

| **Table S2.** Characteristics of the sample of Study two | | | | |
| --- | --- | --- | --- | --- |
| **Size** | **Gender** | **Age** | **STAI-State** | **STAI-Trait** |
| 33 | 18 F/15 M | 23.9± 4.5 y.o SD | 26.6 ± 5.9 SD | 38.7± 9.6 SD |


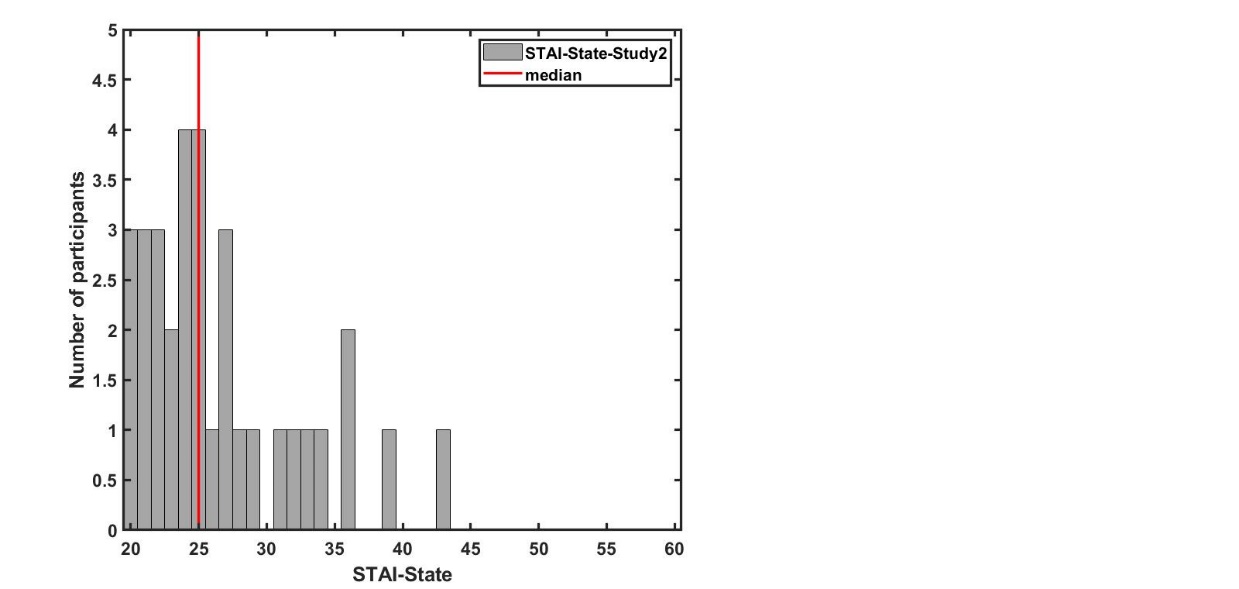

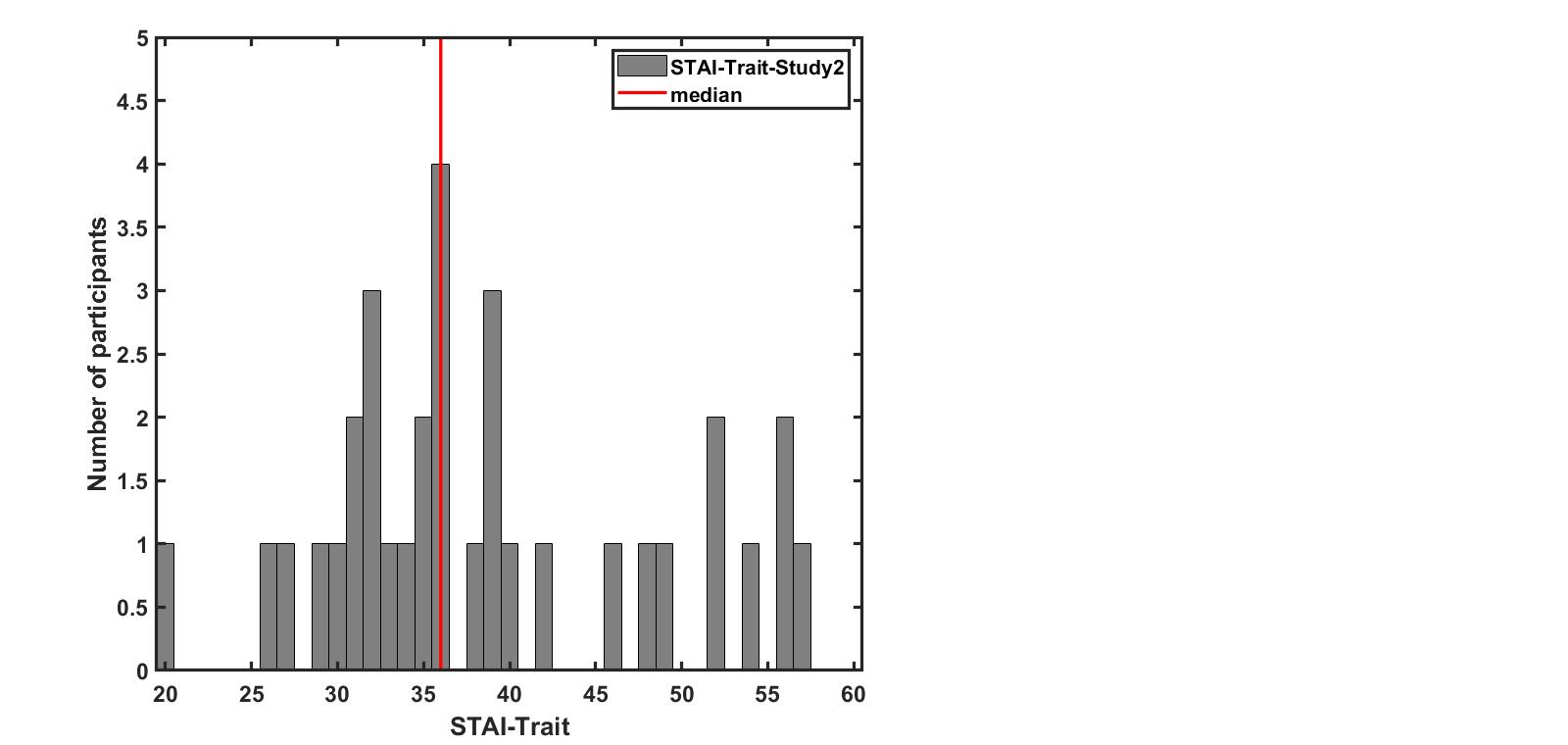


**Figure S2.** Distribution of scores at the STAI-State (Left panel) and STAI-Trait (Right panel) questionnaires of Study one

### Filter quality check

We show skin conductance response to scream (max(RED 1to6s following a scream)-Mean(RED 1s before a scream) for the raw data as well as for the filtered data. Low-pass filter of 0.05Hz appears efficient to reduce the phasic influence of screams (see figure S2 Left). Moreover, and importantly, this filter didn’t affect SCL values. As it can be seen, the values of SCL before and after filter are similar (see figure S2 Right).


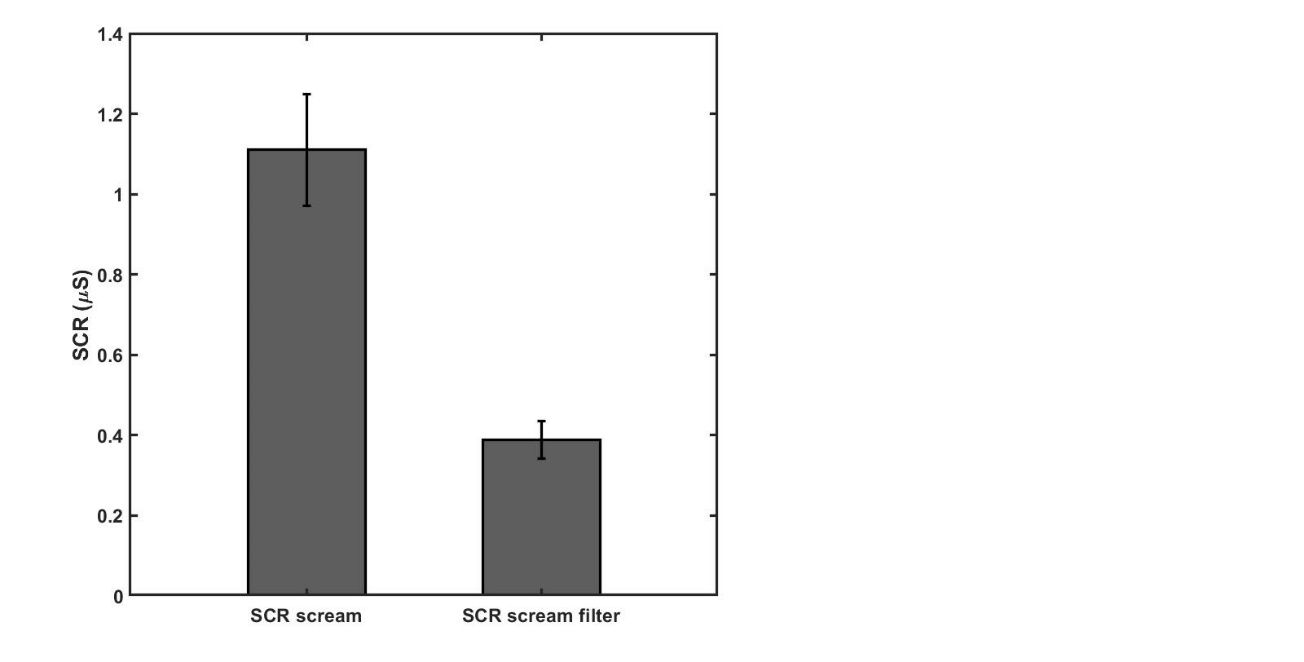

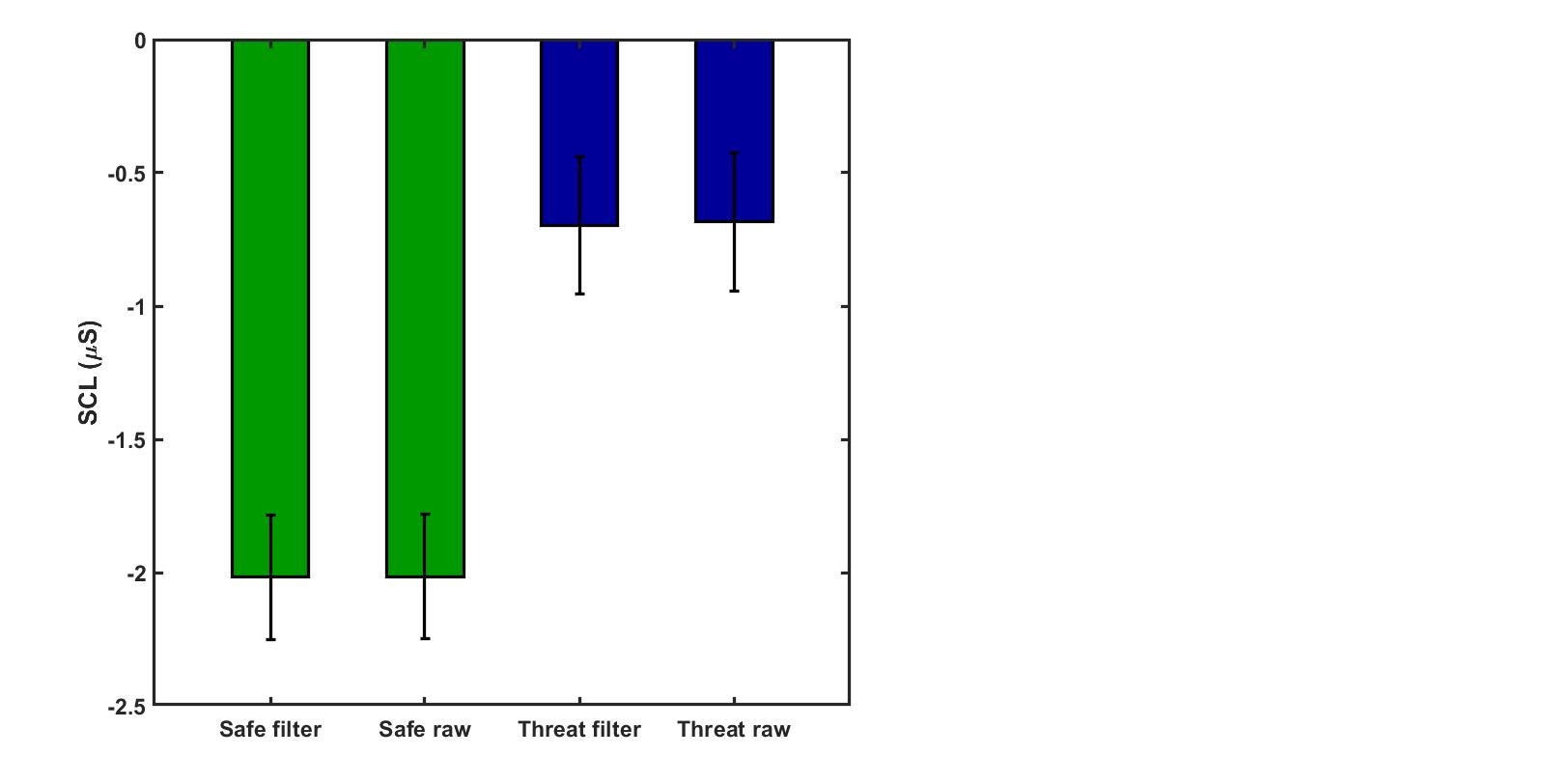


**Figure S3.** SCR to scream (Right) and SCL Before and after filter correction

### Debriefing with participants

We collected ratings for all screams together on an aversive scale (from 0 - not at all aversive to 10 - extremely aversive). Participants rated the screams as moderately aversive (median of 4, see **Figure S4,** left). Inducing anxiety relies both on the aversiveness of the delivered stimuli as well as on their unpredictability. We therefore also asked participants to rate how much they felt preoccupied by the possibility that a scream would be delivered during threat blocks (this was done during the debriefing). Participants felt relatively preoccupied, i.e. they reported anticipatory anxiety of scream delivery (median of 6 see **Figure S4,** right).


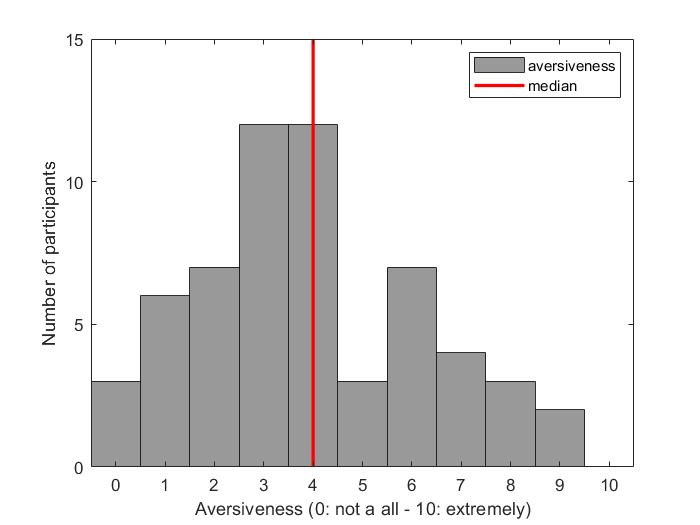

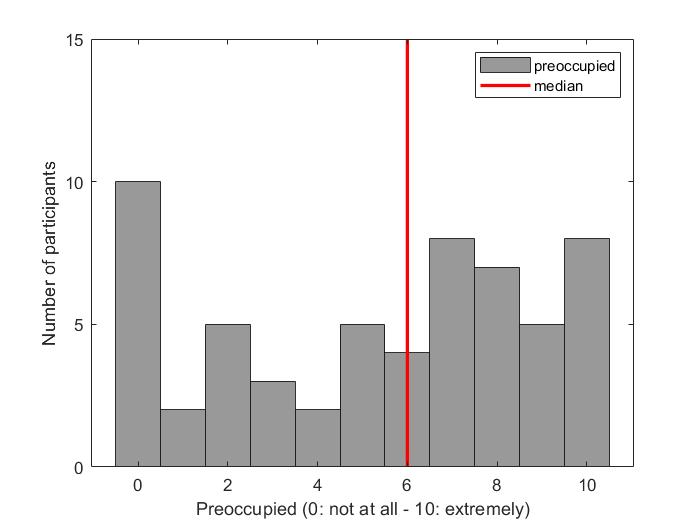


**Figure S4**. Distribution of participant’s aversive (Left) and preoccupation ratings (Right).

# Experiment 1 – Supplementary results

### Skin Conductance Level

**
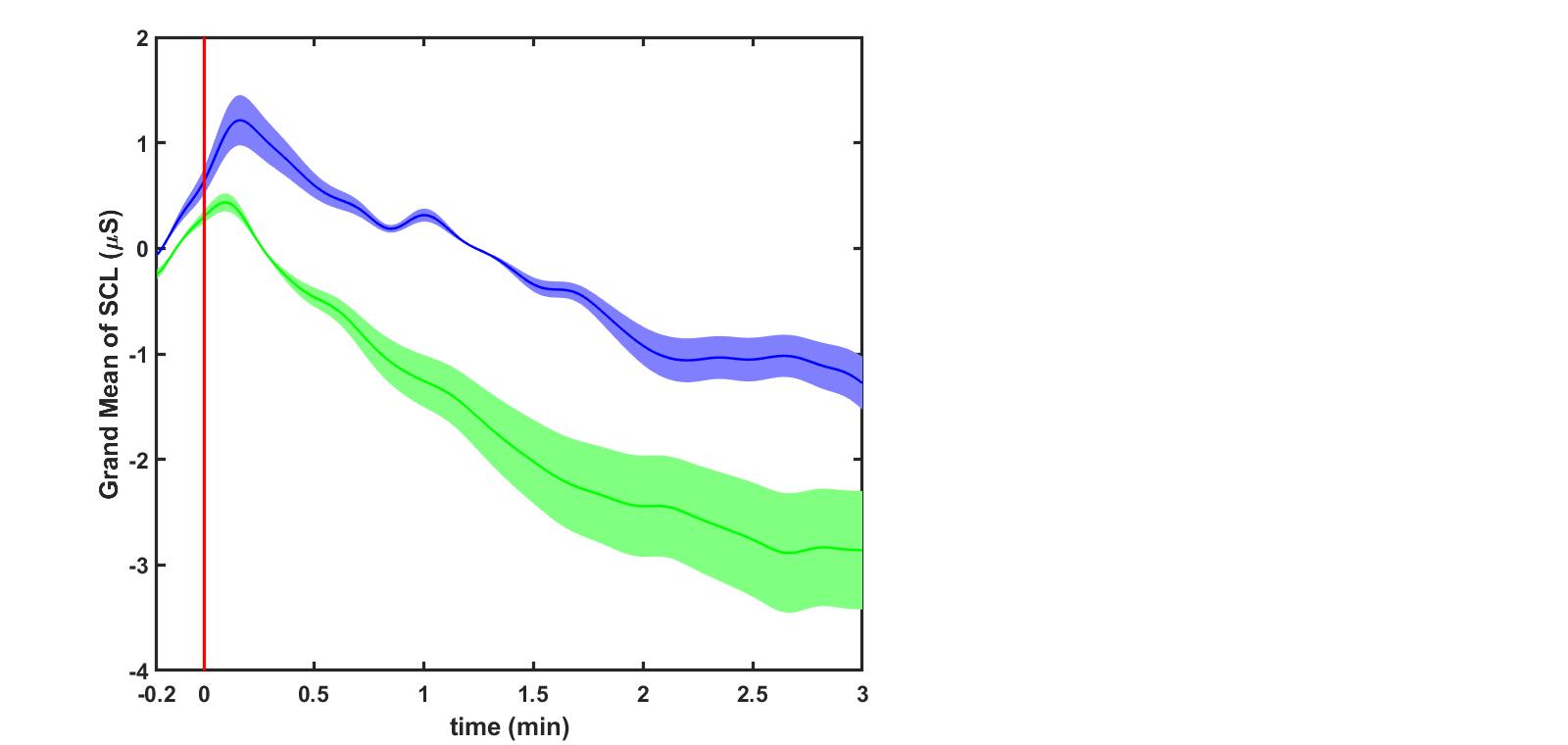
**

**Figure S5.** Grand mean of skin conductance activity across the duration of one block, for threat (blue curve) and safe (green curve) conditions separately. Shaded error bars indicate SEM. At t = -0.2 min, the type of block (“Threat of Safe”) was announced. At t = 0 (red line), the decision-task began.

| **Table S3.** Within Subjects Effects of Repeated Measures ANOVA (Condition & Time) on SCL | | | | | | | | | | | | | | | |
| --- | --- | --- | --- | --- | --- | --- | --- | --- | --- | --- | --- | --- | --- | --- | --- |
|  | | **Sphericity Correction** | | **Sum of Squares** | | **df** | | **Mean Square** | | **F** | | **p** | | **η² _p_** | |
| Condition |  | None |  | 132.517 |  | 1.000 |  | 132.517 |  | 17.204 |  | < .001 |  | 0.408 |  |
|  |  | Greenhouse-Geisser |  | 132.517 |  | 1.000 |  | 132.517 |  | 17.204 |  | < .001 |  | 0.408 |  |
| Residual |  | None |  | 192.565 |  | 25.000 |  | 7.703 |  |  |  |  |  |  |  |
|  |  | Greenhouse-Geisser |  | 192.565 |  | 25.000 |  | 7.703 |  |  |  |  |  |  |  |
| Time |  | None |  | 372.006 | ᵃ | 4.000 | ᵃ | 93.002 | ᵃ | 12.958 | ᵃ | < .001 | ᵃ | 0.341 |  |
|  |  | Greenhouse-Geisser |  | 372.006 | ᵃ | 1.972 | ᵃ | 188.634 | ᵃ | 12.958 | ᵃ | < .001 | ᵃ | 0.341 |  |
| Residual |  | None |  | 717.709 |  | 100.000 |  | 7.177 |  |  |  |  |  |  |  |
|  |  | Greenhouse-Geisser |  | 717.709 |  | 49.303 |  | 14.557 |  |  |  |  |  |  |  |
| Condition ✻ Time |  | None |  | 3.528 | ᵃ | 4.000 | ᵃ | 0.882 | ᵃ | 0.115 | ᵃ | 0.977 | ᵃ | 0.005 |  |
|  |  | Greenhouse-Geisser |  | 3.528 | ᵃ | 2.324 | ᵃ | 1.518 | ᵃ | 0.115 | ᵃ | 0.917 | ᵃ | 0.005 |  |
| Residual |  | None |  | 769.397 |  | 100.000 |  | 7.694 |  |  |  |  |  |  |  |
|  |  | Greenhouse-Geisser |  | 769.397 |  | 58.088 |  | 13.245 |  |  |  |  |  |  |  |
|  | | | | | | | | | | | | | | | |
| Note.  Type III Sum of Squares | | | | | | | | | | | | | | | |
| ᵃ Mauchly's test of sphericity indicates that the assumption of sphericity is violated (p < .05). | | | | | | | | | | | | | | | |

| **Table S4.** Test of Sphericity for Repeated Measures ANOVA (Condition & Time) on SCL | | | | | | | | | | | | | | | |
| --- | --- | --- | --- | --- | --- | --- | --- | --- | --- | --- | --- | --- | --- | --- | --- |
|  | | **Mauchly's W** | | **Approx. Χ²** | | **df** | | **p** | | **Greenhouse-Geisser ε** | | **Huynh-Feldt ε** | | **Lower Bound ε** | |
| Condition |  | 1.000 | ᵃ | NaN | ᵃ | NaN | ᵃ | NaN | ᵃ | 1.000 | ᵃ | 1.000 | ᵃ | 1.000 | ᵃ |
| Time |  | 0.130 |  | 47.783 |  | 9 |  | < .001 |  | 0.493 |  | 0.535 |  | 0.250 |  |
| Condition ✻ Time |  | 0.164 |  | 42.337 |  | 9 |  | < .001 |  | 0.581 |  | 0.644 |  | 0.250 |  |
|  | | | | | | | | | | | | | | | |
| ᵃ Singular error SSP matrix: The repeated measure has only two levels, or more levels than observations. When the repeated measure has two levels, the assumption of sphericity is always met. | | | | | | | | | | | | | | | |

| **Table S5.** Descriptive Statistics (ANOVA) - SCL | | | | | | | | | |
| --- | --- | --- | --- | --- | --- | --- | --- | --- | --- |
| **Condition** | | **Time** | | **Mean** | | **SD** | | **N** | |
| Threat |  | B1 |  | 1.740 |  | 3.533 |  | 26 |  |
|  |  | B2 |  | -0.791 |  | 2.448 |  | 26 |  |
|  |  | B3 |  | -1.003 |  | 2.876 |  | 26 |  |
|  |  | B4 |  | -1.035 |  | 2.224 |  | 26 |  |
|  |  | B5 |  | -1.943 |  | 2.783 |  | 26 |  |
| Safe |  | B1 |  | 0.245 |  | 4.755 |  | 26 |  |
|  |  | B2 |  | -2.590 |  | 2.475 |  | 26 |  |
|  |  | B3 |  | -2.457 |  | 2.184 |  | 26 |  |
|  |  | B4 |  | -2.334 |  | 2.771 |  | 26 |  |
|  |  | B5 |  | -3.036 |  | 3.512 |  | 26 |  |
|  | | | | | | | | | |

| **Table S6.** Post Hoc Comparisons - ANOVA (Time) on SCL_Threat - SCL_Safe | | | | | | | | | | | | | |
| --- | --- | --- | --- | --- | --- | --- | --- | --- | --- | --- | --- | --- | --- |
|  | |  | | **Mean Difference** | | **SE** | | **t** | | **Cohen's d** | | **p _bonf_** | |
| Threat_B1-Safe_B1 |  | Threat_B2-Safe_B2 |  | -0.304 |  | 1.361 |  | -0.223 |  | -0.044 |  | 1.000 |  |
|  |  | Threat_B3-Safe_B3 |  | 0.041 |  | 1.457 |  | 0.028 |  | 0.005 |  | 1.000 |  |
|  |  | Threat_B4-Safe_B4 |  | 0.196 |  | 1.428 |  | 0.137 |  | 0.027 |  | 1.000 |  |
|  |  | Threat_B5-Safe_B5 |  | 0.401 |  | 1.418 |  | 0.283 |  | 0.056 |  | 1.000 |  |
| Threat_B2-Safe_B2 |  | Threat_B3-Safe_B3 |  | 0.345 |  | 0.713 |  | 0.484 |  | 0.095 |  | 1.000 |  |
|  |  | Threat_B4-Safe_B4 |  | 0.500 |  | 0.466 |  | 1.073 |  | 0.210 |  | 1.000 |  |
|  |  | Threat_B5-Safe_B5 |  | 0.706 |  | 0.735 |  | 0.960 |  | 0.188 |  | 1.000 |  |
| Threat_B3-Safe_B3 |  | Threat_B4-Safe_B4 |  | 0.155 |  | 0.911 |  | 0.170 |  | 0.033 |  | 1.000 |  |
|  |  | Threat_B5-Safe_B5 |  | 0.361 |  | 1.019 |  | 0.354 |  | 0.069 |  | 1.000 |  |
| Threat_B4-Safe_B4 |  | Threat_B5-Safe_B5 |  | 0.205 |  | 0.823 |  | 0.250 |  | 0.049 |  | 1.000 |  |
|  | | | | | | | | | | | | | |
| *Note.*  Cohen's d does not correct for multiple comparisons. | | | | | | | | | | | | | |
| *Note.*  Bonferroni adjusted confidence intervals. | | | | | | | | | | | | | |

| **Table S7.** Descriptive Statistics (ANOVA) on SCL_Threat - SCL_Safe | | | | | | | |
| --- | --- | --- | --- | --- | --- | --- | --- |
| **TimeXcondition** | | **Mean** | | **SD** | | **N** | |
| Threat_B1-Safe_B1 |  | 1.495 |  | 6.479 |  | 26 |  |
| Threat_B2-Safe_B2 |  | 1.799 |  | 2.383 |  | 26 |  |
| Threat_B3-Safe_B3 |  | 1.454 |  | 3.355 |  | 26 |  |
| Threat_B4-Safe_B4 |  | 1.299 |  | 2.967 |  | 26 |  |
| Threat_B5-Safe_B5 |  | 1.093 |  | 3.041 |  | 26 |  |
|  | | | | | | | |

| **Table S8.** One Sample T-Test Against 0 for SCL_Threat - SCL_Safe | | | | | | | | | | | | | |
| --- | --- | --- | --- | --- | --- | --- | --- | --- | --- | --- | --- | --- | --- |
|  | | | | | | | | | | **95% CI for Cohen's d** | | | |
|  | | **t** | | **df** | | **p** | | **Cohen's d** | | **Lower** | | **Upper** | |
| Threat_B1-Safe_B1 |  | 1.176 |  | 25 |  | 0.251 |  | 0.231 |  | -0.161 |  | 0.618 |  |
| Threat_B2-Safe_B2 |  | 3.849 |  | 25 |  | < .001 |  | 0.755 |  | 0.312 |  | 1.186 |  |
| Threat_B3-Safe_B3 |  | 2.210 |  | 25 |  | 0.036 |  | 0.433 |  | 0.027 |  | 0.832 |  |
| Threat_B4-Safe_B4 |  | 2.232 |  | 25 |  | 0.035 |  | 0.438 |  | 0.031 |  | 0.837 |  |
| Threat_B5-Safe_B5 |  | 1.833 |  | 25 |  | 0.079 |  | 0.360 |  | -0.041 |  | 0.753 |  |
|  | | | | | | | | | | | | | |
| *Note.*  Student's t-test. | | | | | | | | | | | | | |
| *Note.*  For the Student t-test, effect size is given by Cohen's *d* . | | | | | | | | | | | | | |

### Subjective Anxiety

| **Table S9.** Within Subjects Effects of Repeated Measures ANOVA (Condition & Time) on Subjective Anxiety | | | | | | | | | | | | | | | |
| --- | --- | --- | --- | --- | --- | --- | --- | --- | --- | --- | --- | --- | --- | --- | --- |
|  | | **Sphericity Correction** | | **Sum of Squares** | | **df** | | **Mean Square** | | **F** | | **p** | | **η² _p_** | |
| Condition |  | None |  | 13753.388 |  | 1.000 |  | 13753.388 |  | 15.107 |  | < .001 |  | 0.377 |  |
|  |  | Greenhouse-Geisser |  | 13753.388 |  | 1.000 |  | 13753.388 |  | 15.107 |  | < .001 |  | 0.377 |  |
| Residual |  | None |  | 22760.312 |  | 25.000 |  | 910.412 |  |  |  |  |  |  |  |
|  |  | Greenhouse-Geisser |  | 22760.312 |  | 25.000 |  | 910.412 |  |  |  |  |  |  |  |
| Time |  | None |  | 1046.946 | ᵃ | 4.000 | ᵃ | 261.737 | ᵃ | 1.841 | ᵃ | 0.127 | ᵃ | 0.069 |  |
|  |  | Greenhouse-Geisser |  | 1046.946 | ᵃ | 2.434 | ᵃ | 430.053 | ᵃ | 1.841 | ᵃ | 0.159 | ᵃ | 0.069 |  |
| Residual |  | None |  | 14217.654 |  | 100.000 |  | 142.177 |  |  |  |  |  |  |  |
|  |  | Greenhouse-Geisser |  | 14217.654 |  | 60.862 |  | 233.607 |  |  |  |  |  |  |  |
| Condition ✻ Time |  | None |  | 792.977 |  | 4.000 |  | 198.244 |  | 2.143 |  | 0.081 |  | 0.079 |  |
|  |  | Greenhouse-Geisser |  | 792.977 |  | 2.883 |  | 275.051 |  | 2.143 |  | 0.105 |  | 0.079 |  |
| Residual |  | None |  | 9252.823 |  | 100.000 |  | 92.528 |  |  |  |  |  |  |  |
|  |  | Greenhouse-Geisser |  | 9252.823 |  | 72.075 |  | 128.377 |  |  |  |  |  |  |  |
|  | | | | | | | | | | | | | | | |
| Note.  Type III Sum of Squares | | | | | | | | | | | | | | | |
| ᵃ Mauchly's test of sphericity indicates that the assumption of sphericity is violated (p < .05). | | | | | | | | | | | | | | | |

| **Table S10.** Test of Sphericity for Repeated Measures ANOVA (Condition & Time) on Subjective Anxiety | | | | | | | | | | | | | | | |
| --- | --- | --- | --- | --- | --- | --- | --- | --- | --- | --- | --- | --- | --- | --- | --- |
|  | | **Mauchly's W** | | **Approx. Χ²** | | **df** | | **p** | | **Greenhouse-Geisser ε** | | **Huynh-Feldt ε** | | **Lower Bound ε** | |
| Condition |  | 1.000 | ᵃ | NaN | ᵃ | NaN | ᵃ | NaN | ᵃ | 1.000 | ᵃ | 1.000 | ᵃ | 1.000 | ᵃ |
| Time |  | 0.240 |  | 33.412 |  | 9 |  | < .001 |  | 0.609 |  | 0.679 |  | 0.250 |  |
| Condition ✻ Time |  | 0.510 |  | 15.778 |  | 9 |  | 0.072 |  | 0.721 |  | 0.825 |  | 0.250 |  |
|  | | | | | | | | | | | | | | | |
| ᵃ Singular error SSP matrix: The repeated measure has only two levels, or more levels than observations. When the repeated measure has two levels, the assumption of sphericity is always met. | | | | | | | | | | | | | | | |

| **Table S11.** Descriptive Statistics (ANOVA) on Subjective Anxiety | | | | | | | | | |
| --- | --- | --- | --- | --- | --- | --- | --- | --- | --- |
| **Condition** | | **Time** | | **Mean** | | **SD** | | **N** | |
| Threat |  | B1 |  | 43.000 |  | 23.091 |  | 26 |  |
|  |  | B2 |  | 39.115 |  | 24.438 |  | 26 |  |
|  |  | B3 |  | 37.692 |  | 23.306 |  | 26 |  |
|  |  | B4 |  | 36.731 |  | 25.373 |  | 26 |  |
|  |  | B5 |  | 32.962 |  | 25.677 |  | 26 |  |
| Safe |  | B1 |  | 22.346 |  | 19.781 |  | 26 |  |
|  |  | B2 |  | 25.808 |  | 20.151 |  | 26 |  |
|  |  | B3 |  | 25.269 |  | 17.676 |  | 26 |  |
|  |  | B4 |  | 20.923 |  | 17.202 |  | 26 |  |
|  |  | B5 |  | 22.423 |  | 17.936 |  | 26 |  |
|  | | | | | | | | | |

| **Table S12.** Post Hoc Comparisons - ANOVA (Time) on Subjective Anxiety_Threat - Subjective Anxiety_Safe | | | | | | | | | | | | | |
| --- | --- | --- | --- | --- | --- | --- | --- | --- | --- | --- | --- | --- | --- |
|  | |  | | **Mean Difference** | | **SE** | | **t** | | **Cohen's d** | | **p _bonf_** | |
| Threat_B1-Safe_B1 |  | Threat_B2-Safe_B2 |  | 7.346 |  | 4.926 |  | 1.491 |  | 0.292 |  | 1.000 |  |
|  |  | Threat_B3-Safe_B3 |  | 8.231 |  | 4.567 |  | 1.802 |  | 0.353 |  | 0.836 |  |
|  |  | Threat_B4-Safe_B4 |  | 4.846 |  | 4.807 |  | 1.008 |  | 0.198 |  | 1.000 |  |
|  |  | Threat_B5-Safe_B5 |  | 10.115 |  | 4.152 |  | 2.436 |  | 0.478 |  | 0.223 |  |
| Threat_B2-Safe_B2 |  | Threat_B3-Safe_B3 |  | 0.885 |  | 3.126 |  | 0.283 |  | 0.056 |  | 1.000 |  |
|  |  | Threat_B4-Safe_B4 |  | -2.500 |  | 3.487 |  | -0.717 |  | -0.141 |  | 1.000 |  |
|  |  | Threat_B5-Safe_B5 |  | 2.769 |  | 2.873 |  | 0.964 |  | 0.189 |  | 1.000 |  |
| Threat_B3-Safe_B3 |  | Threat_B4-Safe_B4 |  | -3.385 |  | 3.149 |  | -1.075 |  | -0.211 |  | 1.000 |  |
|  |  | Threat_B5-Safe_B5 |  | 1.885 |  | 2.890 |  | 0.652 |  | 0.128 |  | 1.000 |  |
| Threat_B4-Safe_B4 |  | Threat_B5-Safe_B5 |  | 5.269 |  | 2.902 |  | 1.816 |  | 0.356 |  | 0.815 |  |
|  | | | | | | | | | | | | | |
| *Note.*  Cohen's d does not correct for multiple comparisons. | | | | | | | | | | | | | |
| *Note.*  Bonferroni adjusted confidence intervals. | | | | | | | | | | | | | |

| **Table S13.** Descriptive Statistics (ANOVA) on Subjective Anxiety_Threat - Subjective Anxiety_Safe | | | | | | | |
| --- | --- | --- | --- | --- | --- | --- | --- |
| **TimeXcondition** | | **Mean** | | **SD** | | **N** | |
| Threat_B1-Safe_B1 |  | 20.654 |  | 26.068 |  | 26 |  |
| Threat_B2-Safe_B2 |  | 13.308 |  | 21.012 |  | 26 |  |
| Threat_B3-Safe_B3 |  | 12.423 |  | 19.265 |  | 26 |  |
| Threat_B4-Safe_B4 |  | 15.808 |  | 22.578 |  | 26 |  |
| Threat_B5-Safe_B5 |  | 10.538 |  | 23.646 |  | 26 |  |
|  | | | | | | | |

| **Table S14.** One Sample T-Test Against 0 for Subjective Anxiety_Threat - Subjective Anxiety_Safe | | | | | | | | | | | | | |
| --- | --- | --- | --- | --- | --- | --- | --- | --- | --- | --- | --- | --- | --- |
|  | | | | | | | | | | **95% CI for Cohen's d** | | | |
|  | | **t** | | **df** | | **p** | | **Cohen's d** | | **Lower** | | **Upper** | |
| Threat_B1-Safe_B1 |  | 4.040 |  | 25 |  | < .001 |  | 0.792 |  | 0.344 |  | 1.228 |  |
| Threat_B2-Safe_B2 |  | 3.229 |  | 25 |  | 0.003 |  | 0.633 |  | 0.206 |  | 1.050 |  |
| Threat_B3-Safe_B3 |  | 3.288 |  | 25 |  | 0.003 |  | 0.645 |  | 0.216 |  | 1.063 |  |
| Threat_B4-Safe_B4 |  | 3.570 |  | 25 |  | 0.001 |  | 0.700 |  | 0.264 |  | 1.125 |  |
| Threat_B5-Safe_B5 |  | 2.273 |  | 25 |  | 0.032 |  | 0.446 |  | 0.038 |  | 0.845 |  |
|  | | | | | | | | | | | | | |
| Note.  Student's t-test. | | | | | | | | | | | | | |
| Note.  For the Student t-test, effect size is given by Cohen's d . | | | | | | | | | | | | | |

### Intra-individual correlation

| **Table S15.** One Sample T-Test Against 0 for Intra-individual Correlation | | | | | | | | | | | | | |
| --- | --- | --- | --- | --- | --- | --- | --- | --- | --- | --- | --- | --- | --- |
|  | | | | | | | | | | **95% CI for Cohen's d** | | | |
|  | | **t** | | **df** | | **p** | | **Cohen's d** | | **Lower** | | **Upper** | |
| r _to_z_-Fisher Normalization |  | 2.856 |  | 25 |  | 0.009 |  | 0.560 |  | 0.141 |  | 0.969 |  |
|  | | | | | | | | | | | | | |
| *Note.*  Student's t-test. | | | | | | | | | | | | | |
| *Note.*  For the Student t-test, effect size is given by Cohen's *d* . | | | | | | | | | | | | | |

| **Table S16.** Descriptive Statistics (t-test) on Intra-individual Correlation | | | | | | | | | |  |
| --- | --- | --- | --- | --- | --- | --- | --- | --- | --- | --- |
|  | | **N** | | **Mean** | | **SD** | | **SE** | |  |
| r _to_z_-Fisher Normalization |  | 26.000 |  | 0.256 |  | | 0.457 |  | 0.090 |  |
|  | | | | | | | | | |  |

### STAI-Trait and Skin conductance level (SCL)

| **Table S17.** Within Subjects Effects of Repeated Measures ANOVA (Condition & Time) on SCL with Z-score STAI-Trait | | | | | | | | | | | | | | | |
| --- | --- | --- | --- | --- | --- | --- | --- | --- | --- | --- | --- | --- | --- | --- | --- |
|  | | **Sphericity Correction** | | **Sum of Squares** | | **df** | | **Mean Square** | | **F** | | **p** | | **η² _p_** | |
| Condition |  | None |  | 1.252 |  | 1.000 |  | 1.252 |  | 0.165 |  | 0.688 |  | 0.007 |  |
|  |  | Greenhouse-Geisser |  | 1.252 |  | 1.000 |  | 1.252 |  | 0.165 |  | 0.688 |  | 0.007 |  |
| Condition ✻ ZST |  | None |  | 10.919 |  | 1.000 |  | 10.919 |  | 1.443 |  | 0.241 |  | 0.057 |  |
|  |  | Greenhouse-Geisser |  | 10.919 |  | 1.000 |  | 10.919 |  | 1.443 |  | 0.241 |  | 0.057 |  |
| Residual |  | None |  | 181.646 |  | 24.000 |  | 7.569 |  |  |  |  |  |  |  |
|  |  | Greenhouse-Geisser |  | 181.646 |  | 24.000 |  | 7.569 |  |  |  |  |  |  |  |
| Time |  | None |  | 5.486 | ᵃ | 4.000 | ᵃ | 1.372 | ᵃ | 0.184 | ᵃ | 0.946 | ᵃ | 0.008 |  |
|  |  | Greenhouse-Geisser |  | 5.486 | ᵃ | 1.970 | ᵃ | 2.784 | ᵃ | 0.184 | ᵃ | 0.829 | ᵃ | 0.008 |  |
| Time ✻ ZST |  | None |  | 3.582 | ᵃ | 4.000 | ᵃ | 0.896 | ᵃ | 0.120 | ᵃ | 0.975 | ᵃ | 0.005 |  |
|  |  | Greenhouse-Geisser |  | 3.582 | ᵃ | 1.970 | ᵃ | 1.818 | ᵃ | 0.120 | ᵃ | 0.884 | ᵃ | 0.005 |  |
| Residual |  | None |  | 714.126 |  | 96.000 |  | 7.439 |  |  |  |  |  |  |  |
|  |  | Greenhouse-Geisser |  | 714.126 |  | 47.292 |  | 15.100 |  |  |  |  |  |  |  |
| Condition ✻ Time |  | None |  | 52.130 | ᵃ | 4.000 | ᵃ | 13.032 | ᵃ | 1.745 | ᵃ | 0.146 | ᵃ | 0.068 |  |
|  |  | Greenhouse-Geisser |  | 52.130 | ᵃ | 2.210 | ᵃ | 23.591 | ᵃ | 1.745 | ᵃ | 0.181 | ᵃ | 0.068 |  |
| Condition ✻ Time ✻ ZST |  | None |  | 52.444 | ᵃ | 4.000 | ᵃ | 13.111 | ᵃ | 1.756 | ᵃ | 0.144 | ᵃ | 0.068 |  |
|  |  | Greenhouse-Geisser |  | 52.444 | ᵃ | 2.210 | ᵃ | 23.734 | ᵃ | 1.756 | ᵃ | 0.180 | ᵃ | 0.068 |  |
| Residual |  | None |  | 716.952 |  | 96.000 |  | 7.468 |  |  |  |  |  |  |  |
|  |  | Greenhouse-Geisser |  | 716.952 |  | 53.032 |  | 13.519 |  |  |  |  |  |  |  |
|  | | | | | | | | | | | | | | | |
| Note.  Type III Sum of Squares | | | | | | | | | | | | | | | |
| ᵃ Mauchly's test of sphericity indicates that the assumption of sphericity is violated (p < .05). | | | | | | | | | | | | | | | |

### STAI-Trait and Subjective anxiety

| **Table S18.** Within Subjects Effects of Repeated Measures ANOVA (Condition & Time) on Subjective Anxiety with Z-score STAI-Trait | | | | | | | | | | | | | | | | | | | |
| --- | --- | --- | --- | --- | --- | --- | --- | --- | --- | --- | --- | --- | --- | --- | --- | --- | --- | --- | --- |
|  | | | | | | **Sphericity Correction** | | **Sum of Squares** | | **df** | | **Mean Square** | | **F** | | **p** | | **η² _p_** | |
| Condition | | | |  | | None |  | 13758.162 |  | 1.000 |  | 13758.162 |  | 14.534 |  | < .001 |  | 0.377 |  |
|  | | | |  | | Greenhouse-Geisser |  | 13758.162 |  | 1.000 |  | 13758.162 |  | 14.534 |  | < .001 |  | 0.377 |  |
| Condition ✻ ZST | | | |  | | None |  | 40.833 |  | 1.000 |  | 40.833 |  | 0.043 |  | 0.837 |  | 0.002 |  |
|  | | | |  | | Greenhouse-Geisser |  | 40.833 |  | 1.000 |  | 40.833 |  | 0.043 |  | 0.837 |  | 0.002 |  |
| Residual | | | |  | | None |  | 22719.478 |  | 24.000 |  | 946.645 |  |  |  |  |  |  |  |
|  | | | |  | | Greenhouse-Geisser |  | 22719.478 |  | 24.000 |  | 946.645 |  |  |  |  |  |  |  |
| Time | | | |  | | None |  | 1167.583 | ᵃ | 4.000 | ᵃ | 291.896 | ᵃ | 2.027 | ᵃ | 0.097 | ᵃ | 0.078 |  |
|  | | | |  | | Greenhouse-Geisser |  | 1167.583 | ᵃ | 2.390 | ᵃ | 488.479 | ᵃ | 2.027 | ᵃ | 0.133 | ᵃ | 0.078 |  |
| Time ✻ ZST | | | |  | | None |  | 392.404 | ᵃ | 4.000 | ᵃ | 98.101 | ᵃ | 0.681 | ᵃ | 0.607 | ᵃ | 0.028 |  |
|  | | | |  | | Greenhouse-Geisser |  | 392.404 | ᵃ | 2.390 | ᵃ | 164.169 | ᵃ | 0.681 | ᵃ | 0.535 | ᵃ | 0.028 |  |
| Residual | | | |  | | None |  | 13825.249 |  | 96.000 |  | 144.013 |  |  |  |  |  |  |  |
|  | | | |  | | Greenhouse-Geisser |  | 13825.249 |  | 57.366 |  | 241.002 |  |  |  |  |  |  |  |
| Condition ✻ Time | | | |  | | None |  | 734.440 |  | 4.000 |  | 183.610 |  | 1.954 |  | 0.108 |  | 0.075 |  |
|  | | | |  | | Greenhouse-Geisser |  | 734.440 |  | 2.810 |  | 261.343 |  | 1.954 |  | 0.133 |  | 0.075 |  |
| Condition ✻ Time ✻ ZST | | | |  | | None |  | 233.913 |  | 4.000 |  | 58.478 |  | 0.622 |  | 0.648 |  | 0.025 |  |
|  | | | |  | | Greenhouse-Geisser |  | 233.913 |  | 2.810 |  | 83.236 |  | 0.622 |  | 0.593 |  | 0.025 |  |
| Residual | | | |  | | None |  | 9018.910 |  | 96.000 |  | 93.947 |  |  |  |  |  |  |  |
|  | | | |  | | Greenhouse-Geisser |  | 9018.910 |  | 67.446 |  | 133.720 |  |  |  |  |  |  |  |
|  | | | | | | | | | | | | | | | | | | | |
| Note.  Type III Sum of Squares | | | | | | | | | | | | | | | | | | | |
| ᵃ Mauchly's test of sphericity indicates that the assumption of sphericity is violated (p < .05). | | | | | | | | | | | | | | | | | | | |
|  |  |  |  | |  |  |  |  |  |  |  |  |  |  |  |  |  |  |  |

### STAI-Trait and Intra-individual correlation

| **Table S19.** Pearson Correlation between intra-individual correlation and Z-score STAI-Trait | | | | | | | |
| --- | --- | --- | --- | --- | --- | --- | --- |
|  | |  | | **r _to_z_-Fisher Normalization** | | **ZST** | |
| r _to_z_-Fisher Normalization |  | Pearson's r |  | — |  |  |  |
|  |  | p-value |  | — |  |  |  |
| ZST |  | Pearson's r |  | -0.103 |  | — |  |
|  |  | p-value |  | 0.617 |  | — |  |
|  | | | | | | | |

# Experiment 2 – Supplementary results

### Skin Conductance Level

| **Table S20.** Within Subjects Effects of Repeated Measures ANOVA (Condition & Time) on SCL | | | | | | | | | | | | | | | |
| --- | --- | --- | --- | --- | --- | --- | --- | --- | --- | --- | --- | --- | --- | --- | --- |
|  | | **Sphericity Correction** | | **Sum of Squares** | | **df** | | **Mean Square** | | **F** | | **p** | | **η² _p_** | |
| Condition |  | None |  | 125.907 |  | 1.000 |  | 125.907 |  | 29.359 |  | < .001 |  | 0.478 |  |
|  |  | Greenhouse-Geisser |  | 125.907 |  | 1.000 |  | 125.907 |  | 29.359 |  | < .001 |  | 0.478 |  |
| Residual |  | None |  | 137.234 |  | 32.000 |  | 4.289 |  |  |  |  |  |  |  |
|  |  | Greenhouse-Geisser |  | 137.234 |  | 32.000 |  | 4.289 |  |  |  |  |  |  |  |
| Time |  | None |  | 183.408 | ᵃ | 4.000 | ᵃ | 45.852 | ᵃ | 12.484 | ᵃ | < .001 | ᵃ | 0.281 |  |
|  |  | Greenhouse-Geisser |  | 183.408 | ᵃ | 2.916 | ᵃ | 62.891 | ᵃ | 12.484 | ᵃ | < .001 | ᵃ | 0.281 |  |
| Residual |  | None |  | 470.124 |  | 128.000 |  | 3.673 |  |  |  |  |  |  |  |
|  |  | Greenhouse-Geisser |  | 470.124 |  | 93.322 |  | 5.038 |  |  |  |  |  |  |  |
| Condition ✻ Time |  | None |  | 17.859 |  | 4.000 |  | 4.465 |  | 1.353 |  | 0.254 |  | 0.041 |  |
|  |  | Greenhouse-Geisser |  | 17.859 |  | 3.338 |  | 5.351 |  | 1.353 |  | 0.259 |  | 0.041 |  |
| Residual |  | None |  | 422.344 |  | 128.000 |  | 3.300 |  |  |  |  |  |  |  |
|  |  | Greenhouse-Geisser |  | 422.344 |  | 106.804 |  | 3.954 |  |  |  |  |  |  |  |
|  | | | | | | | | | | | | | | | |
| Note.  Type III Sum of Squares | | | | | | | | | | | | | | | |
| ᵃ Mauchly's test of sphericity indicates that the assumption of sphericity is violated (p < .05). | | | | | | | | | | | | | | | |

| **Table S21.** Test of Sphericity for Repeated Measures ANOVA (Condition & Time) on SCL | | | | | | | | | | | | | | | |
| --- | --- | --- | --- | --- | --- | --- | --- | --- | --- | --- | --- | --- | --- | --- | --- |
|  | | **Mauchly's W** | | **Approx. Χ²** | | **df** | | **p** | | **Greenhouse-Geisser ε** | | **Huynh-Feldt ε** | | **Lower Bound ε** | |
| Condition |  | 1.000 | ᵃ | NaN | ᵃ | NaN | ᵃ | NaN | ᵃ | 1.000 | ᵃ | 1.000 | ᵃ | 1.000 | ᵃ |
| Time |  | 0.514 |  | 20.264 |  | 9 |  | 0.017 |  | 0.729 |  | 0.810 |  | 0.250 |  |
| Condition ✻ Time |  | 0.641 |  | 13.541 |  | 9 |  | 0.140 |  | 0.834 |  | 0.943 |  | 0.250 |  |
|  | | | | | | | | | | | | | | | |
| ᵃ Singular error SSP matrix: The repeated measure has only two levels, or more levels than observations. When the repeated measure has two levels, the assumption of sphericity is always met. | | | | | | | | | | | | | | | |

| **Table S22.** Descriptive Statistics (ANOVA) on SCL | | | | | | | | | |
| --- | --- | --- | --- | --- | --- | --- | --- | --- | --- |
| **Condition** | | **Time** | | **Mean** | | **SD** | | **N** | |
| Threat |  | B1 |  | 0.683 |  | 1.885 |  | 33 |  |
|  |  | B2 |  | -0.162 |  | 2.366 |  | 33 |  |
|  |  | B3 |  | -0.922 |  | 2.363 |  | 33 |  |
|  |  | B4 |  | -1.787 |  | 2.944 |  | 33 |  |
|  |  | B5 |  | -1.652 |  | 3.433 |  | 33 |  |
| Safe |  | B1 |  | -0.974 |  | 1.956 |  | 33 |  |
|  |  | B2 |  | -1.797 |  | 2.256 |  | 33 |  |
|  |  | B3 |  | -2.316 |  | 2.592 |  | 33 |  |
|  |  | B4 |  | -2.187 |  | 2.754 |  | 33 |  |
|  |  | B5 |  | -2.743 |  | 2.763 |  | 33 |  |
|  | | | | | | | | | |

| **Table S23.** Post Hoc Comparisons - ANOVA (Time) on SCL_Threat - SCL_Safe | | | | | | | | | | | | | |
| --- | --- | --- | --- | --- | --- | --- | --- | --- | --- | --- | --- | --- | --- |
|  | |  | | **Mean Difference** | | **SE** | | **t** | | **Cohen's d** | | **p _bonf_** | |
| Threat_B1-Safe_B1 |  | Threat_B2-Safe_B2 |  | 0.022 |  | 0.485 |  | 0.045 |  | 0.008 |  | 1.000 |  |
|  |  | Threat_B3-Safe_B3 |  | 0.263 |  | 0.694 |  | 0.379 |  | 0.066 |  | 1.000 |  |
|  |  | Threat_B4-Safe_B4 |  | 1.258 |  | 0.661 |  | 1.902 |  | 0.331 |  | 0.662 |  |
|  |  | Threat_B5-Safe_B5 |  | 0.566 |  | 0.716 |  | 0.790 |  | 0.138 |  | 1.000 |  |
| Threat_B2-Safe_B2 |  | Threat_B3-Safe_B3 |  | 0.241 |  | 0.602 |  | 0.401 |  | 0.070 |  | 1.000 |  |
|  |  | Threat_B4-Safe_B4 |  | 1.236 |  | 0.655 |  | 1.886 |  | 0.328 |  | 0.683 |  |
|  |  | Threat_B5-Safe_B5 |  | 0.544 |  | 0.586 |  | 0.929 |  | 0.162 |  | 1.000 |  |
| Threat_B3-Safe_B3 |  | Threat_B4-Safe_B4 |  | 0.994 |  | 0.584 |  | 1.702 |  | 0.296 |  | 0.985 |  |
|  |  | Threat_B5-Safe_B5 |  | 0.303 |  | 0.561 |  | 0.540 |  | 0.094 |  | 1.000 |  |
| Threat_B4-Safe_B4 |  | Threat_B5-Safe_B5 |  | -0.691 |  | 0.736 |  | -0.940 |  | -0.164 |  | 1.000 |  |
|  | | | | | | | | | | | | | |
| *Note.*  Cohen's d does not correct for multiple comparisons. | | | | | | | | | | | | | |
| *Note.*  Bonferroni adjusted confidence intervals. | | | | | | | | | | | | | |

| **Table S24.** Descriptive Statistics (ANOVA) on SCL_Threat - SCL_Safe | | | | | | | |
| --- | --- | --- | --- | --- | --- | --- | --- |
| **TimeXcondition** | | **Mean** | | **SD** | | **N** | |
| Threat_B1-Safe_B1 |  | 1.657 |  | 2.526 |  | 33 |  |
| Threat_B2-Safe_B2 |  | 1.635 |  | 2.587 |  | 33 |  |
| Threat_B3-Safe_B3 |  | 1.394 |  | 2.472 |  | 33 |  |
| Threat_B4-Safe_B4 |  | 0.400 |  | 2.582 |  | 33 |  |
| Threat_B5-Safe_B5 |  | 1.091 |  | 3.021 |  | 33 |  |
|  | | | | | | | |

| **Table S25.** One Sample T-Test Against 0 for SCL_Threat - SCL_Safe | | | | | | | | | | | | | |
| --- | --- | --- | --- | --- | --- | --- | --- | --- | --- | --- | --- | --- | --- |
|  | | | | | | | | | | **95% CI for Cohen's d** | | | |
|  | | **t** | | **df** | | **p** | | **Cohen's d** | | **Lower** | | **Upper** | |
| Threat_B1-Safe_B1 |  | 3.769 |  | 32 |  | < .001 |  | 0.656 |  | 0.275 |  | 1.029 |  |
| Threat_B2-Safe_B2 |  | 3.632 |  | 32 |  | < .001 |  | 0.632 |  | 0.254 |  | 1.003 |  |
| Threat_B3-Safe_B3 |  | 3.239 |  | 32 |  | 0.003 |  | 0.564 |  | 0.192 |  | 0.928 |  |
| Threat_B4-Safe_B4 |  | 0.889 |  | 32 |  | 0.381 |  | 0.155 |  | -0.190 |  | 0.497 |  |
| Threat_B5-Safe_B5 |  | 2.075 |  | 32 |  | 0.046 |  | 0.361 |  | 0.006 |  | 0.711 |  |
|  | | | | | | | | | | | | | |
| Note.  Student's t-test. | | | | | | | | | | | | | |
| Note.  For the Student t-test, effect size is given by Cohen's d . | | | | | | | | | | | | | |

### Subjective Anxiety

| **Table S26.** Within Subjects Effects of Repeated Measures ANOVA (Condition & Time) on Subjective Anxiety | | | | | | | | | | | | | | | |
| --- | --- | --- | --- | --- | --- | --- | --- | --- | --- | --- | --- | --- | --- | --- | --- |
|  | | **Sphericity Correction** | | **Sum of Squares** | | **df** | | **Mean Square** | | **F** | | **p** | | **η² _p_** | |
| Condition |  | None |  | 51113.482 |  | 1.000 |  | 51113.482 |  | 47.844 |  | < .001 |  | 0.599 |  |
|  |  | Greenhouse-Geisser |  | 51113.482 |  | 1.000 |  | 51113.482 |  | 47.844 |  | < .001 |  | 0.599 |  |
| Residual |  | None |  | 34187.018 |  | 32.000 |  | 1068.344 |  |  |  |  |  |  |  |
|  |  | Greenhouse-Geisser |  | 34187.018 |  | 32.000 |  | 1068.344 |  |  |  |  |  |  |  |
| Time |  | None |  | 1900.558 | ᵃ | 4.000 | ᵃ | 475.139 | ᵃ | 2.472 | ᵃ | 0.048 | ᵃ | 0.072 |  |
|  |  | Greenhouse-Geisser |  | 1900.558 | ᵃ | 2.324 | ᵃ | 817.702 | ᵃ | 2.472 | ᵃ | 0.083 | ᵃ | 0.072 |  |
| Residual |  | None |  | 24603.042 |  | 128.000 |  | 192.211 |  |  |  |  |  |  |  |
|  |  | Greenhouse-Geisser |  | 24603.042 |  | 74.377 |  | 330.790 |  |  |  |  |  |  |  |
| Condition ✻ Time |  | None |  | 2116.655 |  | 4.000 |  | 529.164 |  | 4.046 |  | 0.004 |  | 0.112 |  |
|  |  | Greenhouse-Geisser |  | 2116.655 |  | 3.311 |  | 639.242 |  | 4.046 |  | 0.007 |  | 0.112 |  |
| Residual |  | None |  | 16739.345 |  | 128.000 |  | 130.776 |  |  |  |  |  |  |  |
|  |  | Greenhouse-Geisser |  | 16739.345 |  | 105.958 |  | 157.981 |  |  |  |  |  |  |  |
|  | | | | | | | | | | | | | | | |
| *Note.*  Type III Sum of Squares | | | | | | | | | | | | | | | |
| ᵃ Mauchly's test of sphericity indicates that the assumption of sphericity is violated (p < .05). | | | | | | | | | | | | | | | |

| **Table S27.** Test of Sphericity for Repeated Measures ANOVA (Condition & Time) on Subjective Anxiety | | | | | | | | | | | | | | | |
| --- | --- | --- | --- | --- | --- | --- | --- | --- | --- | --- | --- | --- | --- | --- | --- |
|  | | **Mauchly's W** | | **Approx. Χ²** | | **df** | | **p** | | **Greenhouse-Geisser ε** | | **Huynh-Feldt ε** | | **Lower Bound ε** | |
| Condition |  | 1.000 | ᵃ | NaN | ᵃ | NaN | ᵃ | NaN | ᵃ | 1.000 | ᵃ | 1.000 | ᵃ | 1.000 | ᵃ |
| Time |  | 0.291 |  | 37.593 |  | 9 |  | < .001 |  | 0.581 |  | 0.629 |  | 0.250 |  |
| Condition ✻ Time |  | 0.641 |  | 13.547 |  | 9 |  | 0.140 |  | 0.828 |  | 0.935 |  | 0.250 |  |
|  | | | | | | | | | | | | | | | |
| ᵃ Singular error SSP matrix: The repeated measure has only two levels, or more levels than observations. When the repeated measure has two levels, the assumption of sphericity is always met. | | | | | | | | | | | | | | | |

| **Table S28.** Descriptive Statistics (ANOVA) – Subjective Anxiety | | | | | | | | | |
| --- | --- | --- | --- | --- | --- | --- | --- | --- | --- |
| **Condition** | | **Time** | | **Mean** | | **SD** | | **N** | |
| Threat |  | B1 |  | 47.303 |  | 26.789 |  | 33 |  |
|  |  | B2 |  | 41.727 |  | 25.790 |  | 33 |  |
|  |  | B3 |  | 36.303 |  | 26.052 |  | 33 |  |
|  |  | B4 |  | 36.030 |  | 26.687 |  | 33 |  |
|  |  | B5 |  | 34.030 |  | 25.576 |  | 33 |  |
| Safe |  | B1 |  | 14.000 |  | 15.383 |  | 33 |  |
|  |  | B2 |  | 14.364 |  | 13.212 |  | 33 |  |
|  |  | B3 |  | 12.455 |  | 14.992 |  | 33 |  |
|  |  | B4 |  | 15.091 |  | 19.106 |  | 33 |  |
|  |  | B5 |  | 15.030 |  | 19.404 |  | 33 |  |
|  | | | | | | | | | |

| **Table S29.** Post Hoc Comparisons - ANOVA (Time) on Subjective Anxiety_Threat – Subjective Anxiety_Safe | | | | | | | | | | | | | |
| --- | --- | --- | --- | --- | --- | --- | --- | --- | --- | --- | --- | --- | --- |
|  | |  | | **Mean Difference** | | **SE** | | **t** | | **Cohen's d** | | **p _bonf_** | |
| Threat_B1-Safe_B1 |  | Threat_B2-Safe_B2 |  | 5.939 |  | 3.923 |  | 1.514 |  | 0.264 |  | 1.000 |  |
|  |  | Threat_B3-Safe_B3 |  | 9.455 |  | 4.581 |  | 2.064 |  | 0.359 |  | 0.472 |  |
|  |  | Threat_B4-Safe_B4 |  | 12.364 |  | 3.631 |  | 3.405 |  | 0.593 |  | 0.018 |  |
|  |  | Threat_B5-Safe_B5 |  | 14.303 |  | 4.204 |  | 3.402 |  | 0.592 |  | 0.018 |  |
| Threat_B2-Safe_B2 |  | Threat_B3-Safe_B3 |  | 3.515 |  | 3.508 |  | 1.002 |  | 0.174 |  | 1.000 |  |
|  |  | Threat_B4-Safe_B4 |  | 6.424 |  | 2.904 |  | 2.212 |  | 0.385 |  | 0.342 |  |
|  |  | Threat_B5-Safe_B5 |  | 8.364 |  | 4.203 |  | 1.990 |  | 0.346 |  | 0.552 |  |
| Threat_B3-Safe_B3 |  | Threat_B4-Safe_B4 |  | 2.909 |  | 3.995 |  | 0.728 |  | 0.127 |  | 1.000 |  |
|  |  | Threat_B5-Safe_B5 |  | 4.848 |  | 4.980 |  | 0.974 |  | 0.169 |  | 1.000 |  |
| Threat_B4-Safe_B4 |  | Threat_B5-Safe_B5 |  | 1.939 |  | 3.481 |  | 0.557 |  | 0.097 |  | 1.000 |  |
|  | | | | | | | | | | | | | |
| *Note.*  Cohen's d does not correct for multiple comparisons. | | | | | | | | | | | | | |
| *Note.*  Bonferroni adjusted confidence intervals. | | | | | | | | | | | | | |

| **Table S30.** Descriptive Statistics (ANOVA) Subjective Anxiety_Threat – Subjective Anxiety_Safe | | | | | | | |
| --- | --- | --- | --- | --- | --- | --- | --- |
| **TimeXcondition** | | **Mean** | | **SD** | | **N** | |
| Threat_B1-Safe_B1 |  | 33.303 |  | 26.984 |  | 33 |  |
| Threat_B2-Safe_B2 |  | 27.364 |  | 26.535 |  | 33 |  |
| Threat_B3-Safe_B3 |  | 23.848 |  | 22.857 |  | 33 |  |
| Threat_B4-Safe_B4 |  | 20.939 |  | 25.172 |  | 33 |  |
| Threat_B5-Safe_B5 |  | 19.000 |  | 24.384 |  | 33 |  |
|  | | | | | | | |

| **Table S31.** One Sample T-Test Against 0 for Subjective Anxiety_Threat – Subjective Anxiety_Safe | | | | | | | | | | | | | |
| --- | --- | --- | --- | --- | --- | --- | --- | --- | --- | --- | --- | --- | --- |
|  | | | | | | | | | | **95% CI for Cohen's d** | | | |
|  | | **t** | | **df** | | **p** | | **Cohen's d** | | **Lower** | | **Upper** | |
| Threat_B1-Safe_B1 |  | 7.090 |  | 32 |  | < .001 |  | 1.234 |  | 0.774 |  | 1.684 |  |
| Threat_B2-Safe_B2 |  | 5.924 |  | 32 |  | < .001 |  | 1.031 |  | 0.602 |  | 1.450 |  |
| Threat_B3-Safe_B3 |  | 5.994 |  | 32 |  | < .001 |  | 1.043 |  | 0.612 |  | 1.464 |  |
| Threat_B4-Safe_B4 |  | 4.779 |  | 32 |  | < .001 |  | 0.832 |  | 0.430 |  | 1.224 |  |
| Threat_B5-Safe_B5 |  | 4.476 |  | 32 |  | < .001 |  | 0.779 |  | 0.384 |  | 1.165 |  |
|  | | | | | | | | | | | | | |
| *Note.*  Student's t-test. | | | | | | | | | | | | | |
| *Note.*  For the Student t-test, effect size is given by Cohen's *d* . | | | | | | | | | | | | | |

### Intra-individual correlation

| **Table S32.** One Sample T-Test Against 0 for Intra-individual Correlation | | | | | | | | | | | | | |
| --- | --- | --- | --- | --- | --- | --- | --- | --- | --- | --- | --- | --- | --- |
|  | | | | | | | | | | **95% CI for Cohen's d** | | | |
|  | | **t** | | **df** | | **p** | | **Cohen's d** | | **Lower** | | **Upper** | |
| r _to_z_-Fisher Normalization |  | 5.579 |  | 32 |  | < .001 |  | 0.971 |  | 0.551 |  | 1.381 |  |
|  | | | | | | | | | | | | | |
| *Note.*  Student's t-test. | | | | | | | | | | | | | |
| *Note.*  For the Student t-test, effect size is given by Cohen's *d* . | | | | | | | | | | | | | |

| **Table S33.** Descriptive Statistics (t-test) on Intra-individual Correlation | | | | | | | | | |
| --- | --- | --- | --- | --- | --- | --- | --- | --- | --- |
|  | | **N** | | **Mean** | | **SD** | | **SE** | |
| r _to_z_-Fisher Normalization |  | 33.000 |  | 0.402 |  | 0.414 |  | 0.072 |  |
|  | | | | | | | | | |

### STAI-Trait and Skin conductance level (SCL)

| **Table S34.** Within Subjects Effects of Repeated Measures ANOVA (Condition & Time) on SCL with Z-score STAI-Trait | | | | | | | | | | | | | | | |
| --- | --- | --- | --- | --- | --- | --- | --- | --- | --- | --- | --- | --- | --- | --- | --- |
|  | | **Sphericity Correction** | | **Sum of Squares** | | **df** | | **Mean Square** | | **F** | | **p** | | **η² _p_** | |
| Condition |  | None |  | 125.915 |  | 1.000 |  | 125.915 |  | 28.447 |  | < .001 |  | 0.479 |  |
|  |  | Greenhouse-Geisser |  | 125.915 |  | 1.000 |  | 125.915 |  | 28.447 |  | < .001 |  | 0.479 |  |
| Condition ✻ ZST |  | None |  | 0.019 |  | 1.000 |  | 0.019 |  | 0.004 |  | 0.948 |  | 0.000 |  |
|  |  | Greenhouse-Geisser |  | 0.019 |  | 1.000 |  | 0.019 |  | 0.004 |  | 0.948 |  | 0.000 |  |
| Residual |  | None |  | 137.214 |  | 31.000 |  | 4.426 |  |  |  |  |  |  |  |
|  |  | Greenhouse-Geisser |  | 137.214 |  | 31.000 |  | 4.426 |  |  |  |  |  |  |  |
| Time |  | None |  | 183.481 | ᵃ | 4.000 | ᵃ | 45.870 | ᵃ | 12.270 | ᵃ | < .001 | ᵃ | 0.284 |  |
|  |  | Greenhouse-Geisser |  | 183.481 | ᵃ | 2.878 | ᵃ | 63.762 | ᵃ | 12.270 | ᵃ | < .001 | ᵃ | 0.284 |  |
| Time ✻ ZST |  | None |  | 6.567 | ᵃ | 4.000 | ᵃ | 1.642 | ᵃ | 0.439 | ᵃ | 0.780 | ᵃ | 0.014 |  |
|  |  | Greenhouse-Geisser |  | 6.567 | ᵃ | 2.878 | ᵃ | 2.282 | ᵃ | 0.439 | ᵃ | 0.717 | ᵃ | 0.014 |  |
| Residual |  | None |  | 463.557 |  | 124.000 |  | 3.738 |  |  |  |  |  |  |  |
|  |  | Greenhouse-Geisser |  | 463.557 |  | 89.205 |  | 5.197 |  |  |  |  |  |  |  |
| Condition ✻ Time |  | None |  | 17.892 |  | 4.000 |  | 4.473 |  | 1.345 |  | 0.257 |  | 0.042 |  |
|  |  | Greenhouse-Geisser |  | 17.892 |  | 3.340 |  | 5.357 |  | 1.345 |  | 0.262 |  | 0.042 |  |
| Condition ✻ Time  ✻ ZST |  | None |  | 9.804 |  | 4.000 |  | 2.451 |  | 0.737 |  | 0.569 |  | 0.023 |  |
|  |  | Greenhouse-Geisser |  | 9.804 |  | 3.340 |  | 2.935 |  | 0.737 |  | 0.546 |  | 0.023 |  |
| Residual |  | None |  | 412.540 |  | 124.000 |  | 3.327 |  |  |  |  |  |  |  |
|  |  | Greenhouse-Geisser |  | 412.540 |  | 103.545 |  | 3.984 |  |  |  |  |  |  |  |
|  | | | | | | | | | | | | | | | |
| Note.  Type III Sum of Squares | | | | | | | | | | | | | | | |
| ᵃ Mauchly's test of sphericity indicates that the assumption of sphericity is violated (p < .05). | | | | | | | | | | | | | | | |

### STAI-Trait and Subjective anxiety

| **Table S35.** Within Subjects Effects of Repeated Measures ANOVA (Condition & Time) on Subjective Anxiety with Z-score STAI-Trait | | | | | | | | | | | | | | | |
| --- | --- | --- | --- | --- | --- | --- | --- | --- | --- | --- | --- | --- | --- | --- | --- |
|  | | **Sphericity Correction** | | **Sum of Squares** | | **df** | | **Mean Square** | | **F** | | **p** | | **η² _p_** | |
| Condition |  | None |  | 51185.115 |  | 1.000 |  | 51185.115 |  | 50.981 |  | < .001 |  | 0.622 |  |
|  |  | Greenhouse-Geisser |  | 51185.115 |  | 1.000 |  | 51185.115 |  | 50.981 |  | < .001 |  | 0.622 |  |
| Condition ✻ ZST |  | None |  | 3063.006 |  | 1.000 |  | 3063.006 |  | 3.051 |  | 0.091 |  | 0.090 |  |
|  |  | Greenhouse-Geisser |  | 3063.006 |  | 1.000 |  | 3063.006 |  | 3.051 |  | 0.091 |  | 0.090 |  |
| Residual |  | None |  | 31124.012 |  | 31.000 |  | 1004.000 |  |  |  |  |  |  |  |
|  |  | Greenhouse-Geisser |  | 31124.012 |  | 31.000 |  | 1004.000 |  |  |  |  |  |  |  |
| Time |  | None |  | 1900.320 | ᵃ | 4.000 | ᵃ | 475.080 | ᵃ | 2.403 | ᵃ | 0.053 | ᵃ | 0.072 |  |
|  |  | Greenhouse-Geisser |  | 1900.320 | ᵃ | 2.319 | ᵃ | 819.478 | ᵃ | 2.403 | ᵃ | 0.090 | ᵃ | 0.072 |  |
| Time ✻ ZST |  | None |  | 92.347 | ᵃ | 4.000 | ᵃ | 23.087 | ᵃ | 0.117 | ᵃ | 0.976 | ᵃ | 0.004 |  |
|  |  | Greenhouse-Geisser |  | 92.347 | ᵃ | 2.319 | ᵃ | 39.823 | ᵃ | 0.117 | ᵃ | 0.915 | ᵃ | 0.004 |  |
| Residual |  | None |  | 24510.695 |  | 124.000 |  | 197.667 |  |  |  |  |  |  |  |
|  |  | Greenhouse-Geisser |  | 24510.695 |  | 71.887 |  | 340.961 |  |  |  |  |  |  |  |
| Condition ✻ Time |  | None |  | 2116.067 |  | 4.000 |  | 529.017 |  | 4.024 |  | 0.004 |  | 0.115 |  |
|  |  | Greenhouse-Geisser |  | 2116.067 |  | 3.227 |  | 655.741 |  | 4.024 |  | 0.008 |  | 0.115 |  |
| Condition ✻ Time  ✻ ZST |  | None |  | 437.334 |  | 4.000 |  | 109.333 |  | 0.832 |  | 0.508 |  | 0.026 |  |
|  |  | Greenhouse-Geisser |  | 437.334 |  | 3.227 |  | 135.524 |  | 0.832 |  | 0.487 |  | 0.026 |  |
| Residual |  | None |  | 16302.012 |  | 124.000 |  | 131.468 |  |  |  |  |  |  |  |
|  |  | Greenhouse-Geisser |  | 16302.012 |  | 100.037 |  | 162.961 |  |  |  |  |  |  |  |
|  | | | | | | | | | | | | | | | |
| *Note.*  Type III Sum of Squares | | | | | | | | | | | | | | | |
| ᵃ Mauchly's test of sphericity indicates that the assumption of sphericity is violated (p < .05). | | | | | | | | | | | | | | | |

### STAI-Trait and Intra-individual correlation

| **Table S36.** Pearson Correlation between intra-individual correlation and Z-score STAI-Trait | | | | | | | |
| --- | --- | --- | --- | --- | --- | --- | --- |
|  | |  | | **r _to_z_-Fisher Normalization** | | **ZST** | |
| r _to_z_-Fisher Normalization |  | Pearson's r |  | — |  |  |  |
|  |  | p-value |  | — |  |  |  |
| ZST |  | Pearson's r |  | -0.020 |  | — |  |
|  |  | p-value |  | 0.913 |  | — |  |
|  | | | | | | | |
